# ILDR1 deficiency causes degeneration of cochlear outer hair cells and disrupts the structure of the organ of Corti: a mouse model for human DFNB42

**DOI:** 10.1242/bio.201410876

**Published:** 2015-03-27

**Authors:** Qing Sang, Wen Li, Yao Xu, Ronggui Qu, Zhigang Xu, Ruizhi Feng, Li Jin, Lin He, Huawei Li, Lei Wang

**Affiliations:** 1 State Key Laboratory of Genetic Engineering and MOE Key Laboratory of Contemporary Anthropology, School of Life Sciences, Fudan University, Shanghai, 200032, PR China; 2 Institute of Biomedical Sciences, Fudan University, No 138 Yixueyuan Road, Shanghai, 200032, PR China; 3 Department of Otolaryngology, Eye & ENT hospital, Fudan University, 83 Fenyang Road, Shanghai, 200031, PR China; 4 Bio-X Center, Key Laboratory for the Genetics of Developmental and Neuropsychiatric Disorders, Ministry of Education, Shanghai Jiao Tong University, Shanghai, 200030, PR China; 5 School of Life Sciences, Shandong University, Shandong, China

**Keywords:** ILDR1, Deafness, Genetics

## Abstract

Immunoglobulin-like domain containing receptor 1 (*ILDR1*) is a poorly characterized gene that was first identified in lymphoma cells. Mutations in *ILDR1* are responsible for DFNB42, but the pathogenesis of hearing loss caused by *ILDR1* mutations remains to be elucidated. To explore the role of ILDR1 in hearing, we created *Ildr1* knockout mice. In heterozygous mice, ILDR1 expression was found in outer hair cells (OHCs) and inner hair cells (IHCs) of the organ of Corti. ILDR1-deficient mice are profoundly deaf by postnatal day 21 (P21). No significant difference was observed in the supporting cells and IHCs of ILDR1-deficient mice, but progressive degeneration of OHCs occurred at P15 and disruption of the tunnel running through the organ of Corti was noticeable at P21. By P28, there were no OHCs visible in any of the turns of the organ of Corti, and the tunnel of the organ of Corti was entirely destroyed. ILDR1 deficiency affects expression of tricellulin *in vivo*, and this provides a possible explanation to hearing loss. To further elucidate the mechanism of deafness related to ILDR1 deficiency, we pursued a differential proteomic approach to comprehensively assess differential protein expression in the cochleae of *Ildr1*^+/−^ and *Ildr1*^−/−^ mice at P21. Altogether, 708 proteins were up-regulated (fold change >1.5) and 114 proteins were down-regulated (fold change <0.5) in the *Ildr1*^−/−^ mice compared with *Ildr1*^+/−^ mice. Gene ontology classification indicated that a number of differentially expressed proteins are involved in cell adhesion, protein and vesicle-mediated transport, cell death, membrane organization, and cellular homeostasis. A few of these proteins are closely related to hearing development. Taken together, our data suggest that ILDR1 is important for the survival of OHCs and provide novel insights into the pathogenesis of human deafness DFNB42 deafness.

## INTRODUCTION

Immunoglobulin-like domain containing receptor 1 (*ILDR1*) is a poorly characterized gene that was first identified in lymphoma cells. Protein structure analysis indicates that it is a member of the protein family containing an Ig-like extracellular N-terminal domain and a cysteine-rich region in the C-terminal intracellular domain (Hauge et al., 2004). It is expressed in a variety of organs, including the prostate, testes, pancreas, and kidney. ILDR1 shows approximately 30% homology to lipolysis stimulated receptor, and there is evidence that ILDR1 plays a role in mediating fat-stimulated cholecystokinin secretion (Hauge et al., 2004; Chandra et al., 2013). In addition, overexpression of ILDR1 might have biological implications in myelodysplastic syndromes (Zagaria et al., 2012).

Recently, Borck et al. found both nonsense and frameshift mutations in *ILDR1* in consanguineous Pakistani and Iranian families that are responsible for human DFNB42, a locus for autosomal recessive hearing loss (Borck et al., 2011). In situ hybridization detected expression of *Ildr1* early in the development of the vestibule, hair cells, and supporting cells of the mouse cochlea (Borck et al., 2011). In addition, ILDR1 has been shown to be localized at tricellular contacts and to recruit tricellulin to tricellular contacts in epithelial cells (Higashi et al., 2013). ILDR1 mutant proteins are defective in recruiting tricellulin to tricellular contacts *in vitro*, and tricellulin deficiency has been shown to affect tight junction architecture and cochlear hair cells in mouse models (Higashi et al., 2013; Nayak et al., 2013). Taken together, these data suggest that functional ILDR1 might be essential for hearing development. However, up until now there have been no reports on the hearing phenotype of ILDR1-deficient mice. The function of ILDR1 in hearing is largely unknown, and the molecular mechanisms of ILDR1 in deafness remain to be elucidated.

In this study, we describe the generation of *Ildr1* knockout mice and the results of auditory function tests and histological and molecular experiments. Our results show that deafness related to ILDR1 deficiency is due to degeneration of cochlear outer hair cells (OHCs), the destruction of the tunnel of the organ of Corti, and abnormal expression of tricellulin and other hearing-related proteins. We propose a possible pathological mechanism leading to hearing loss caused by ILDR1 deficiency in DFNB42 patients.

## RESULTS

### Generation of *Ildr1* knockout mice

**Fig. 1.**
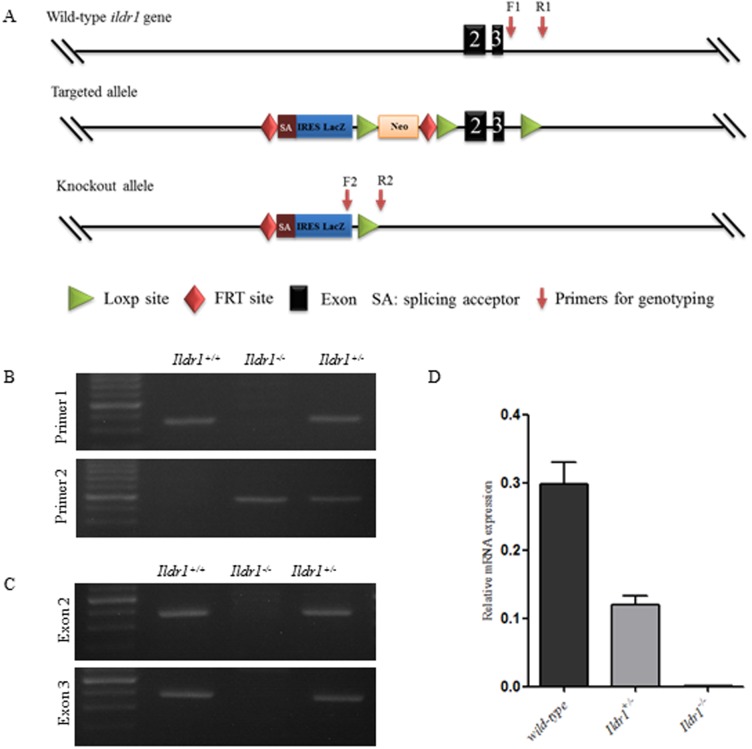
Generation of the *Ildr1* knockout allele and the *Ildr1*^−/−^ mouse. (A) The structure of the wild-type gene and the general features of the targeted allele and knockout allele. Primer pairs for genotyping were indicated by red arrows. (B) Mouse genotyping using RT-PCR. Primer 1 and primer 2 were used to distinguish the wild type allele and knockout allele respectively. (C) RT-PCR shows the deletion of exon 2 and exon 3 in *Ildr1*^−/−^ mouse. (D) qRT-PCR shows the absence of *ildr1* mRNA.

**Fig. 2.**
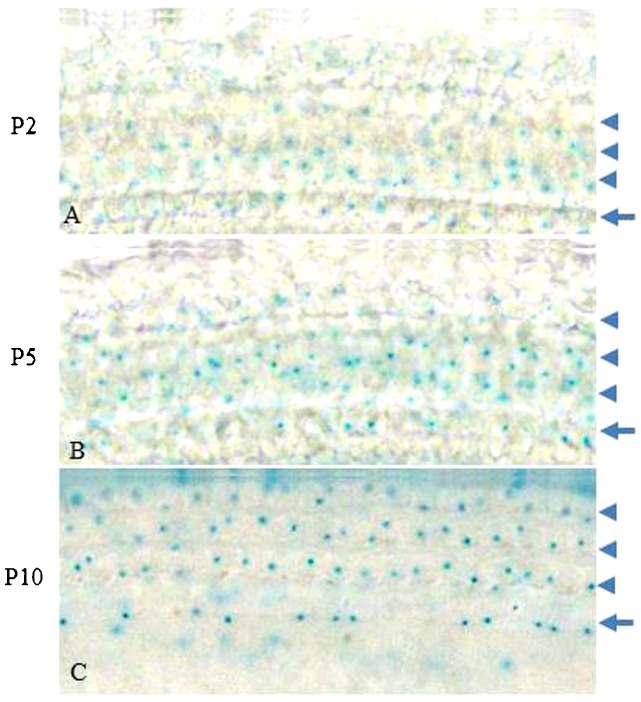
X-gal staining for beta-galactosidase activity in the organ of Corti in P2 (A), P5 (B), and P10 (C) *Ildr1*^+/−^ mice. Arrowheads in A–C point to the three lines of outer hair cells and arrows in A–C point to the single line of inner hair cells.

*Ildr1*, the gene encoding ILDR1, is located on chromosome 16. It has eight exons and encodes the entire protein of 537 amino acids. A targeting vector was constructed and used to put a neomycin cassette and the *LacZ* reporter gene in the upstream region of exon 2 (Fig. 1A). The LacZ-Neo cassette serves as a positive selection marker in the ES targeting step and as the reporter for exploring the expression pattern of ILDR1 (Fig. 2). Exon 2 and exon 3 were floxed by loxp site. The targeted mouse were then mated with B6.C-Tg (CMV-cre)1Cgn mouse to delete exon 2 and exon 3 of gene *Ildr1*. The knockout efficiency was evaluated through the detection of exon 2 and exon 3 using RT-PCR. The resulted indicated that exon 2 and exon 3 were deleted from *Ildr1*^−/−^ mice (Fig. 1C). Mice were genotyped by two PCR primers. The first primer was used to identify the wild-type allele (a single 365 bp band) and the second primer was used to identify the knockout allele (a single 514 bp band) (Fig. 1B).

### *Ildr1* expression pattern in postnatal mice

LacZ expression in the *Ildr1*^+/−^ mice was regulated by the promoter of *Ildr1*, and this allowed the ILDR1 expression pattern to be determined by measuring β-galactosidase activity. β-galactosidase activity was detected by X-gal staining in the basilar membrane from P2, P5, and P10 mice. As shown in Fig. 2, ILDR1 was mainly expressed in the organ of Corti including three lines of outer hair cells (OHCs) and a single line of inner hair cells (IHCs). This expression pattern is consistent with results from previous findings from RNA in situ hybridization experiments (Borck et al., 2011) and results from immunofluorescence with ILDR1 antibody which revealed the expression of ILDR1 in tricellular tight junctions of the organ of Corti (Higashi et al., 2013; Nayak et al., 2013).

### *Ildr1*^−/−^ mice suffer profound hearing impairment by P21

**Fig. 3.**
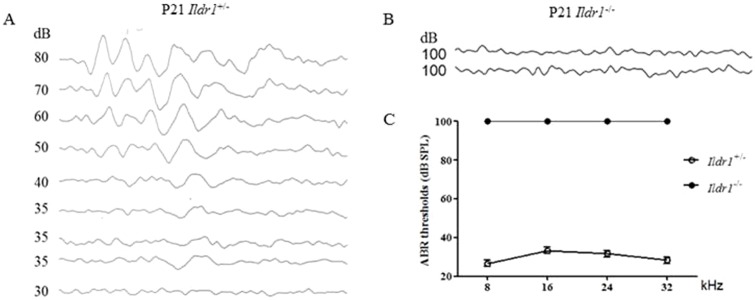
Auditory dysfunction in *Ildr1*^−/−^ mouse. Offspring at P21 derived from *Ildr1*^+/−^ × *Ildr1*^−/−^ mating were subjected to auditory-evoked brainstem response (ABR) testing. (A,B) ABR response to tone bursts at a representative frequency of 8 kHz in P21 *Ildr1*^+/−^ mouse (A) and P21 *Ildr1*^−/−^ mouse (B). (C) The average click sound pressure level (SPL) threshold of *Ildr1*^+/−^ and *Ildr1*^−/−^ mice at P21. *Ildr1*^+/−^ mice showed normal SPL thresholds at all frequencies measured, but *Ildr1*^−/−^ mice showed no waveforms at intensities up to 100 dB SPL at P21 (n = 4 mice for each time point).

To determine if *Idlr1* knockout mice also exhibited a hearing impairment phenotype that mimicked the phenotype of DFNB42, we measured the ABR hearing thresholds in *Ildr1*^+/−^ and *Ildr1*^−/−^ mice at P21. Compared with the *Ildr1*^+/−^ mice, *Ildr1*^−/−^ mice at P21 did not respond to any tone bursts (≥100 dB) demonstrating profound hearing impairment at all frequencies (Fig. 3A–C).

### OHCs in *ILDR1*^−/−^ mice degenerate and the tunnel of the organ of Corti is completely destroyed by P28

**Fig. 4.**
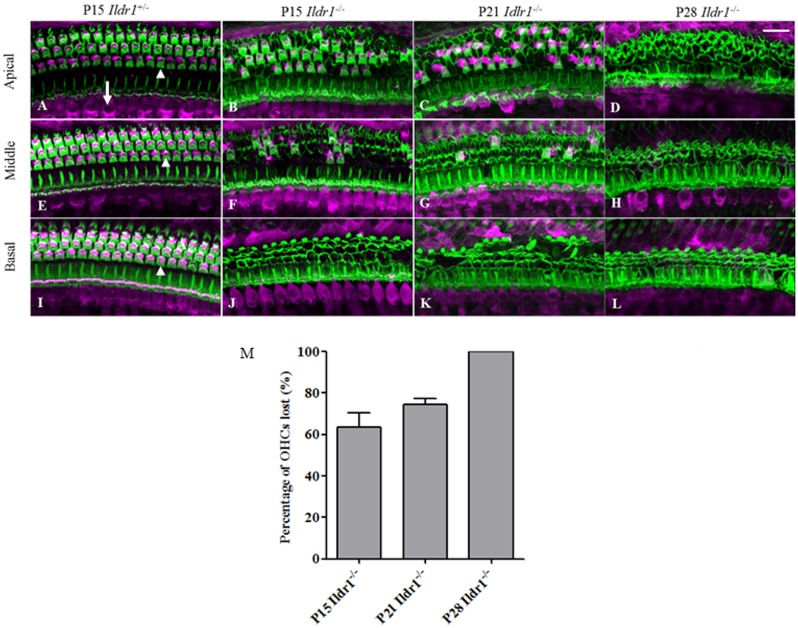
Hair cells in the organ of Corti labeled with myosin 7a antibody (magenta) and counterstained with phalloidin labeling of cytoskeletal filamentous actin (green) in P15, P21, and P28 mice. Hair cells in the apical (A–D), middle (E–H), and basal turns (I–L) of the cochlea are shown separately. (M) Histograms showing the percentage of outer hair cells degenerated in P15, P21, and P28 *Ildr1*^−/−^ mice. Arrowhead in A points to the outer hair cells (OHCs) and arrow in A points to the inner hair cells (IHCs). Scale bar: 20 µm.

**Fig. 5.**
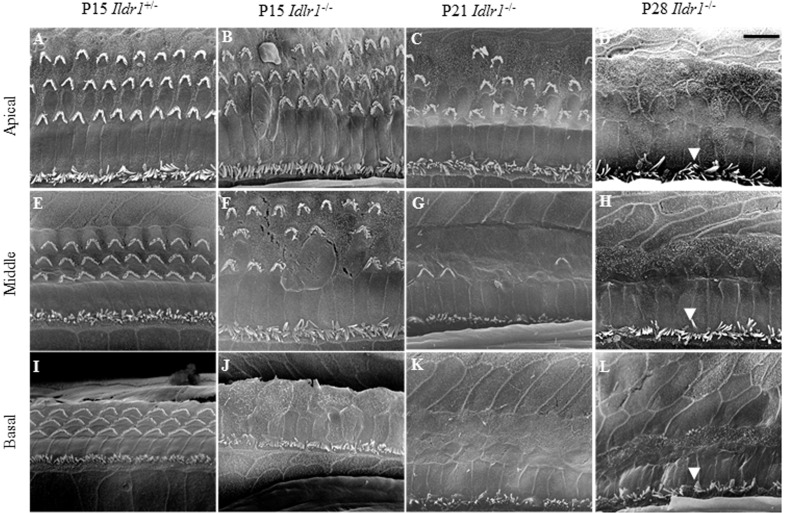
Scanning electron micrographs of cochlear hair cells within the organ of Corti of P15, P21, and P28 mice. Apical (A–D), middle (E–H), and basal turns (I–L) of the cochleae from *Ildr1*^+/−^ and *Ildr1*^−/−^ mice were scanned separately. Bold arrowheads in D,H,L point to the stereocilia of the inner hair cells (IHC), which are normal in *Ildr1*^−/−^ mice. Scale bar: 10 µm.

**Fig. 6.**
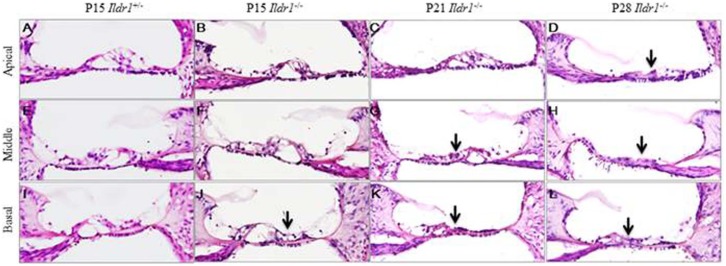
Hematoxylin and eosin labeling of frozen cochlear sections showing the structure of the organ of Corti. The organ of Corti from the apical (A–D), middle (E–H), and basal turns (I–L) of the cochlea are shown separately. Black arrows in J show the disruption of the organ of Corti in the basal turn of P15 *Ildr1*^−/−^ mice. Black arrows in G,K show the progressive disruption of the organ of Corti in the basal and middle turns of P21 *Ildr1*^−/−^ mice. Black arrows in D,H,L show the complete disruption of the organ of Corti in all turns of the cochleae of P28 *Ildr1*^−/−^ mice.

To explore the underlying cause of hearing impairment, the morphologies of OHCs, IHCs, and the organs of Corti were examined at different postnatal developmental stages. Confocal microscopy, scanning electron microscopy, and immunohistochemistry revealed that OHCs, IHCs, supporting cells, and the organs of Corti developed normally and no difference in morphology was seen between P7 *Ildr1*^−/−^ mice and P7 *Ildr1*^+/−^ mice (supplementary material Figs S1 and S2). However, by P15 different degrees of OHC degeneration were observed in the basal, middle, and apical turns of the organ of Corti and approximately 60% of the outer hair cells in the whole cochlea were lost (Fig. 4B,F,J,M). By P21, the degree of OHC degeneration in the three turns of the organ of Corti was more severe than at P15. At P21, no OHCs were seen in the basal turns, there were almost no OHCs left in the middle turns, and only a few OHCs were seen in the apical turns (Fig. 4C,G,K,M). By P28, there were no OHCs visible in any of the turns of the organ of Corti (Fig. 4D,H,L). The immunofluorescence results were confirmed by scanning electron microscopy examination of the morphology of the basilar membrane from P15, P21, and P28 *Ildr1*^+/−^ and *Ildr1*^+/−^ mice (Fig. 5). In addition, hematoxylin and eosin staining showed that from P15 the structure and morphology of the organ of Corti was partly disrupted in the basal turn due to the loss of OHCs (Fig. 6J). The basal and middle turns of the organ of Corti were even more disrupted at P21 (Fig. 6C,G,K). At P28, the organ of Corti was completely disrupted throughout the entire cochlea and this resulted in the disappearance of the tunnel of the organ of Corti (Fig. 6D,H,L). Although OHCs and the organ of Corti showed abnormalities during early postnatal development, IHCs in *Ildr1*^−/−^ mice remained intact throughout the observation period (Figs 4 and 5).

### ILDR1 deficiency decreased the expression of tricellulin in tight junctions *in vivo*

**Fig. 7.**
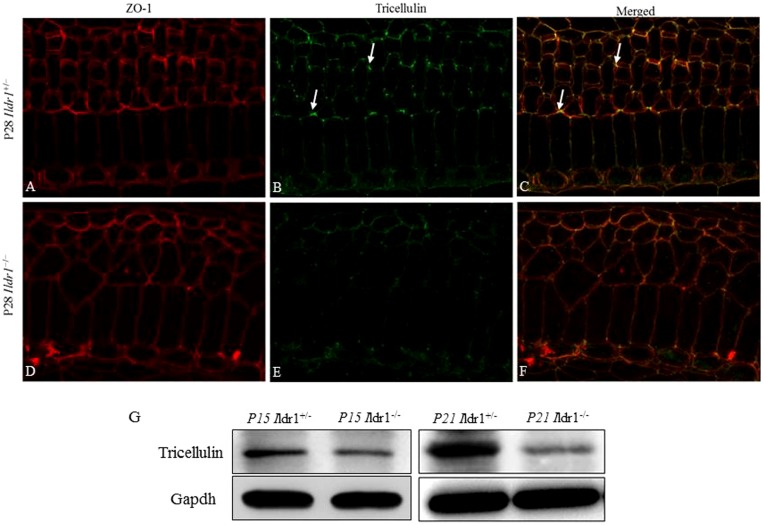
Expression of tricellulin in tight junctions was decreased in *Ildr1*^−/−^ mice. (A–F) Immunofluorescence of the organ of Corti from the middle turn of P28 mice using tricellulin and ZO-1 antibody. Arrows in B,C show the localization of tricellulin in the tricellular tight junctions. (G) Western blot analysis of tricellulin in the basilar membrane at P15 and P21 *Ildr1*^+/−^ and *Ildr1*^−/−^ mice. Gapdh was used as a loading control.

Because ILDR1 has been showed to be localized at the tricellular tight junctions in the organ of Corti of mice and to play a role in the recruitment of tricellulin in epithelial cells (Higashi et al., 2013), we sought to determine if tricellulin is altered in *Ildr1*^−/−^ mice. As shown by immunofluorescence, tricellulin located at the tricellular tight junctions was significantly decreased in the cochleae of *Ildr1*^−/−^ mice compared with the *Ildr1*^+/−^ mice (Fig. 7C,F). This was confirmed by western blot with basilar membrane proteins using tricellulin antibody at P15 and P21 mice (Fig. 7G). It indicates that hearing loss caused by ILDR1 deficiency might be through the abnormal expression of tricellulin.

### Identification of differentially expressed proteins and biological pathway analysis

**Fig. 8.**
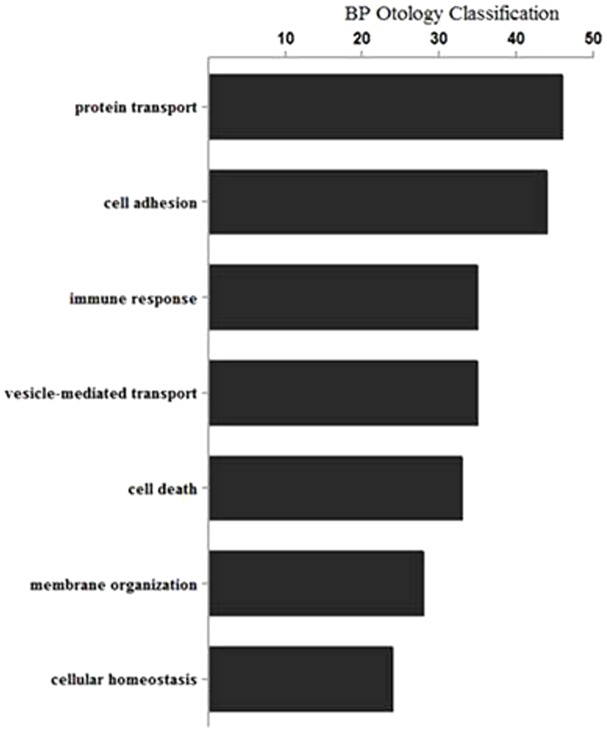
Gene ontology analysis of differentially expressed proteins in the cochleae of *Ildr1*^+/−^ and *Ildr1*^−/−^ P21 mice using the online DAVID software. The x-axis shows the number of proteins in each category.

To further elucidate the mechanism of deafness related to ILDR1 deficiency, we used a differential proteomic approach (LC-MS/MS) to comprehensively assess differential protein expression in the cochleae of *Ildr1*^+/−^ and *Ildr1*^−/−^ mice at P21. We used 70 µg of mouse cochlear protein for LC-MS/MS analysis, and three replicates were run for each individual sample. Only proteins detected in at least two replicates were used in further analysis. Altogether, 708 proteins were up-regulated (fold change >1.5) and 114 proteins were down-regulated (fold change <0.5) in *Ildr1*^−/−^ mice (supplementary material Tables S1, S2). To interpret the likely roles of differentially expressed proteins in the pathology of deafness, we analyzed and built the biological pathways related to these proteins. Identified GO processes were divided into several categories, including cell adhesion, protein and vesicle-mediated transport, cell death, immune response, membrane organization, and cellular homeostasis (Fig. 8).

## DISCUSSION

This study described the pathophysiology of DFNB42, a recessive nonsyndromic form of deafness that is associated with nonsense and frameshift mutations in *ILDR1*. We generated *Ildr1*^−/−^ mice in which ILDR1 was absent in all organs, and this allowed us to study the effects of ILDR1 deficiency *in vivo*. Similar to the phenotype of DFNB42 patients, the homozygote *Ildr1*^−/−^ mice displayed profound hearing loss suggesting the essential role of ILDR1 in hearing development in mammals. When we are preparing the manuscript, Morozko et al. published the *ILDR1* null mouse model, which show the similar phenotypes as ours (Morozko et al., 2015)

We observed expression of ILDR1 in hair cells in the organ of Corti of P2, P5, and P10 mice (Fig. 2) and found that ILDR1 was highly expressed in auditory sensory cells and regions indicating that ILDR1 might have a functional role in hearing. Compared with *Ildr1*^+/−^ mice, no significant differences were observed in supporting cells or IHCs of *Ildr1*^−/−^ mice during early developmental stages. In P7 *Ildr1*^−/−^ mice, OHCs and the organ of Corti were also normal (supplementary material Figs S1 and S2). We found that the loss of ILDR1 resulted in progressive degeneration of OHCs and abnormalities in the tunnel of the organ of Corti that were observable starting at P15. OHCs in the *Ildr1*^−/−^ mice began to degenerate in the basal turns of the organ of Corti from around P15, and as time went on the degeneration of OHCs began to occur in other turns of the organ of Corti. Finally, all OHCs in the basal, middle, and apical turns of the organ of Corti were lost around P28. The tunnel of the organ of Corti showed the same trend as OHCs during the early development period and was entirely destroyed over the course of our observations. These phenotypes in *Ildr1*^−/−^ mice do not appear until P15, and this suggests that ILDR1 is not necessary for the development of hair cells or the establishment of hearing but is important for the survival and maintenance of hearing-related cells.

The molecular mechanisms by which ILDR1 deficiency leads to degeneration of OHCs and related phenotypes remains to be elucidated. Tricellular tight junctions are where three epithelial cells meet, and they play a crucial role in the epithelial barrier function (Anderson and Van Itallie, 2009; Schneeberger and Lynch, 2004). It has been demonstrated that tricellulin and ILDR1 are components of tricellular tight junctions of epithelial cells *in vitro* as well as in the mouse organ of Corti *in vivo* (Higashi et al., 2013). ILDR1 mutant proteins are defective in recruiting tricellulin in epithelial cells, and this implies that ILDR1-mediated recruitment of tricellulin to tricellular tight junctions might be required for hearing (Higashi et al., 2013). However, whether ILDR1 will affect tricellulin recruitment *in vivo* is unknown.

Tricellulin-deficient mice develop rapidly progressing hearing loss accompanied by loss of mechanosensory cochlear hair cells (Nayak et al., 2013). So we investigated tricellulin expression in *Ildr1*^−/−^ mice. As shown in Fig. 7, the protein level of tricellulin in P15 and P21 mice was decreased in *Ildr1*^−/−^ mice compared to *Ildr1*^+/−^ mice. This is the first direct evidence that ILDR1 is functionally associated with tricellulin and that ILDR1 deficiency affects the expression of tricellulin *in vivo*. These results are consistent with previous results showing that ILDR1 affects the recruitment and localization of tricellulin to tricellular junctions in epithelial cells (Higashi et al., 2013) and suggest that profound hearing loss by ILDR1 deficiency might be partly explained by abnormal expression of tricellulin.

In *Tricellulin* deficiency mice, both OHCs and IHCs undergo rapid degeneration (Nayak et al., 2013). In *Ildr1*^−/−^ mice, however, only OHCs and not IHCs undergo progressive degeneration during early postnatal development. One possible explanation for this difference is that IHCs might express other proteins that can compensate for the lack of ILDR1. This phenotype in which only OHCs but not IHCs are affected was also observed in other deafness related gene knockout mice (Ben-Yosef et al., 2003). Another possible explanation for this phenomenon is suggested by evidence that tricellulin deficiency alters the microenvironment of the endolymph and that it affects the regulation of paracellular transport of small uncharged molecules (Nayak et al., 2013). In our study, ILDR1 deficiency affects tricellulin expression. However, because the membranes of IHCs are not surrounded by a liquid-filled space, IHCs might not be sensitive to abnormal concentrations of paracellular small molecules, such as ATP, that might result from abnormal expression of tricellulin in *Ildr1* knockout mice.

Hair cells and organ of Corti structures in *Ildr1*^−/−^ mice develop normally up to two weeks of age indicating that hearing loss is not the result of developmental abnormalities in these mice but rather a result of disruption of homeostasis. To provide a deeper understanding of the molecular changes induced by the ILDR1 deficiency, and to explore the molecular mechanisms of cochlear hair cell degeneration and related phenotypes in *Ildr1*^−/−^ mice, we performed proteomics analysis of the cochleae from *Ildr1*^−/−^ mice. The DAVID bioinformatics tool was used to map the genes that were differentially regulated in *Ildr1*^−/−^ mice into GO categories. GO classification indicated that a number of differentially expressed proteins were involved in cell adhesion, protein and vesicle-mediated transport, immune responses, cell death, membrane organization, and cellular homeostasis (Fig. 8). Several studies suggest that cell adhesion abnormalities and the occurrence of cell death are key features in the pathology of deafness (Zheng and Hu, 2012; Zheng et al., 2011; Excoffon et al., 2006; Kitajiri et al., 2004; Kwon et al., 2014; May et al., 2013; Cohen-Salmon et al., 2002; Cheng et al., 2005).

Among down-regulated proteins in the *Ildr1*^−/−^ mice, some have a close relationship with hearing development and deafness. For example, transient receptor potential cation channel subfamily V member 4 (Trpv4) mediates the calcium influx that is crucial for the hearing process. It has been demonstrated that Ca^2+^ modulates the tip links that are essential for the movement of the stereocilia bundles (Giacomello et al., 2012). There are also a few mitochondria-related proteins that are down-regulated in the cochleae of *Ildr1*^−/−^ mice, including NADH dehydrogenase (ubiquinone) iron-sulfur protein 8 (Ndufs8), mitochondrial calcium uptake 2 (Micu2), mitochondrial 2,4-dienoyl-CoA reductase (Decr1), and mitochondrial carrier homolog 1 (Mtch1). Mitochondrial dysfunction might result in the reduction of ATP production in IHCs and, therefore, to insufficient energy for the OHCs to amplify sound waves (Olmos et al., 2011). Thus, although the exact molecular mechanism of how ILDR1 deficiency results in deafness remains to be elucidated and needs to be further investigated, the differentially expressed proteins described above that might affect hearing by impairing the corresponding pathophysiological pathways suggest possible mechanisms of hearing loss in ILDR1-deficient mice.

In summary, we produced *Ildr1* knockout mice and characterized the subsequent hearing-related phenotypes and molecular mechanisms behind these phenotypes, including apoptotic OHC degeneration, disruption of the tunnel of the organ of Corti, abnormal expression of tricellulin, and profound proteomic changes, all of which resulted in profound hearing loss.

## MATERIALS AND METHODS

### *Ildr1* gene knockout

A 24-kb targeting vector harboring the SA-IRES-LacZ-Neo cassette was constructed containing the floxed *Ildr1* exons 2 and 3 and their flanking sequences. The LacZ-Neo cassette served as a positive selection marker in the ES (embryonic stem cell) targeting step and as the reporter for exploring the expression pattern of ILDR1. The targeting vector was linearized and introduced into the W4 ES cell line by electroporation, and the targeted cell line was screened with long-range PCR. Primers for 3′-end screening were 5′-TATGATCGGAATTGGGCTGCAG-3′ and 5′-TCAGTATTAAGGAATTAGGACCTT-3′. Primers for 5′-end screening were 5′-ATCATTTAGAAACTGCAACCTCTTT-3′ and 5′-CCAACTGACCTTGGGCAAGAACAT-3′. Finally, two selected clones were injected into blastocysts to generate chimeras. Homozygous targeted mouse were then mated with B6.C-Tg(CMV-cre)1Cgn mouse to delete exon 2 and exon 3 of gene *Ildr1*. The final knockout allele and wild type allele were genotyped by PCR of tail-snip DNA with the following two primer pairs respectively: F1: 5′-AGCAAGGGATTCCCCTGATCTTC-3′; R1: 5′-GCAGTGGTCTCAAAGAAGCCACTTG-3′, F2: 5′-CCGGTCG CTACCATTACCAGT-3′; R2: 5′-CGTTGGTATAGGTATGAAGAATGG-3′. Fudan University approved the study.

### ABR measurements

Hearing ability was measured by auditory brainstem response (ABR) analysis at postnatal day P21 in four mice of both the *Ildr1*^+/−^ and *Ildr1*^−/−^ genotypes. The ABR response measures the hearing nerve's response to sounds. Mice were anesthetized with intraperitoneal injections of 5 mg/ml Avertin (2 µl/g body weight, Sigma-Aldrich). All recordings were made in a sound-attenuated chamber using an auditory-evoked potential diagnostic system (Intelligent Hearing Systems) with a high-frequency transducer. Responses to 8, 16, 24, and 32 kHz tone bursts were recorded. Thresholds were determined in 5 dB or 10 dB steps of decreasing stimulus intensity until the waveforms lost their reproducible morphology. The maximum sound intensity tested for each frequency was a 100 dB sound pressure level (SPL).

### X-gal staining

The organ of Corti from P2, P5, and P10 *Ildr1*^+/−^ mice were stained as whole mount. In brief, the basilar membrane were fixed for 20 min at room temperature [1% formaldehyde, 0.2% glutaraldehyde and 0.02% Nonidet P-40 in phosphate-buffered saline (PBS)], washed twice (2 mM MgCl_2_ and 0.02% Nonidet P-40 in PBS) and stained overnight at 37°C (0.1 M MgCl_2_, 5 mM potassium ferricyanide and 1 mg/ml X-gal in PBS). Finally, images were captured using an Olympus BX51 microscope.

### Immunostaining

To view the structure of the hair cells and stereocilia of the organ of Corti, immunostaining and phalloidin staining was performed using a myosin 7a antibody and phalloidin, respectively. The cochleae were dissected from P15, P21, and P28 mice, fixed in 4% paraformaldehyde in PBS for 2 h at room temperature, and decalcified in 16.8% EDTA (pH 7.3) at 4°C for different periods of time according to the age of the mouse. The basilar membrane from the basal, middle, and apical turns of the cochlea was dissected followed by blocking and permeabilization in 0.01 M PBS containing 0.5% Triton X-100 and 10% sheep serum for 1 h at 37°C. The basilar membrane was then incubated with myosin 7a antibody (Proteus BioSciences) diluted to 1:250 in 0.01 M PBS containing 0.1% Triton X-100 and 1% sheep serum for 1 h at 37°C and then at 4°C overnight. The next day, the basilar membrane was washed three times in PBS and then incubated in DyLight 649-conjugated anti-rabbit antibody at 1:500 dilution for 1 h at 37°C. Finally, the basilar membrane was stained with phalloidin in PBS for 1 h at 37°C. For tight junction labeling, the inner ears extracted from mice were fixed 5% trichloroacetic acid (TCA) for 20 min, followed by 3 washes with PBS. The cochlear coils were dissected into apical, middle, and basal pieces and permeabilized in 0.2% Triton X-100 for 20 min, before incubating in blocking buffer (5% BSA, 2% normal goat serum in PBS) for 1 h. The tissue samples were probed with primary ZO-1 antibody (1:200 dilution) and tricellulin (1:200 dilution) antibody (Life Technologies) at 4°C over night. After three washes in PBS, the samples were probed with the secondary antibody for 1 h at room temperature and then washed for three times in PBS. Images were captured using a TCS SP5 confocal laser-scanning microscope (Leica, Germany).

### Scanning electron microscopy

For the scanning electron microscopy analysis, the inner ears were removed from the animals after decapitation and the cochleae were exposed. A small piece of bone was removed from the cochlear apex, and the entire bulla was immersed in fixative (2.5% glutaraldehyde, 0.1 M sodium cacodylate, and 2 mM CaCl_2_ for 1.5 h). After three quick washes with 0.1 M sodium cacodylate buffer, the inner ears were post-fixed in 1% osmium tetroxide in 0.1 M sodium cacodylate buffer for 1 h at room temperature. The inner ears were washed three times with PBS buffer before incubating in PBS containing 0.25 M EDTA for 2 days at 4°C. The samples were then finely dissected in water to remove the stria vascularis, and the spiral ligament was cut off to expose the organ of Corti. The cochlear tissues were then dehydrated in acetone, critical point dried, sputter coated with gold, and imaged on a scanning electron microscope.

### HE staining

Cochleae were dissected from P15, P21, and P28 *Ildr1*^+/−^ and *Ildr1*^−/−^ mice, fixed in 4% paraformaldehyde in PBS for 2 h at room temperature, and decalcified in 16.8% EDTA (pH 7.3) at 4°C over different periods of time according to the age of the mouse. Decalcified cochleae were immersed in 15% sucrose for 2 h, in 30% sucrose for 6 h, and in OCT (Optimum Cutting Temperature) compound at room temperature overnight. The cochleae were sectioned in 10 µm thicknesses with a cryostat microtome. Sections were immersed in PBS for 10 min, stained with hematoxylin for 6 min, and stained with eosin for 18 s. Images were captured using an Olympus BX51 microscope.

### Western blots

Basilar membranes from the organ of Corti were collected from *Ildr1*^+/−^ and *Ildr1*^−/−^ mice in cold lysis buffer [150 mM NaCl, 0.05 M Tris, 1% Triton X-100, and protease inhibitor cocktail (Roche)]. The tissues were homogenized in 1.5-ml tubes with a plastic pestle and sonicated. The lysates were centrifuged at 21,000 ***g*** for 20 min at 4°C, and the supernatants were collected in fresh tubes and mixed with reducing sample buffer and boiled for 5 min. The samples were centrifuged at 21,000 ***g*** for 5 min before loading on a 10% SDS-PAGE gel (Invitrogen). Western blots were carried out using iBlot (Invitrogen), and the proteins were transferred onto nitrocellulose membranes. The membrane blots were blocked with 5% milk for at least 2 h before probing with rabbit anti-tricellulin polyclonal antibody (Life Technologies, catalog no. 48-8400; 1:200 dilution) and rabbit anti-GAPDH polyclonal antibody (Abmart, catalog no. P30008; 1:500 dilution) overnight at 4°C. Blots were probed with goat anti-rabbit IgG-HRP secondary antibody (Abmart, catalog no. M21002; 1:1000 dilution).

### LC-MS/MS analysis

Total proteins were extracted from the cochleae of five *Ildr1*^+/−^ mice and five *Ildr1*^−/−^ mice at P21. SDS-PAGE electrophoresis was used to check the quality of the protein (supplementary material Fig. S3). In brief, a Nano-LC MS/MS experiment was performed on an HPLC system composed of two LC-20AD nano-flow liquid chromatography pumps and one LC-20AB micro-flow LC pump (all from Shimadzu Corporation, Tokyo, Japan) connected to an LTQ-Orbitrap mass spectrometer (Thermo Electron Corporation, San Jose, CA). Seventy micrograms of protein were injected via an SIL-20 AC auto-sampler and loaded onto a CAPTRAP column (0.5 mm × 2 mm, MICHROM Bioresources Inc., Auburn, CA) for 5 min at a flow rate of 60 µl/min. The sample was subsequently separated by a PICOFRIT C18 reverse-phase column (0.075 mm × 100 mm, New Objective Inc., Woburn, MA) at a flow rate of 500 nl/min. The separated sample was introduced into the mass spectrometer via a 15 µm silica tip (New Objective Inc.) adapted to a DYNAMIC nano-electrospray source (Thermo Electron Corporation). The mass spectrometer was operated in data-dependent mode and each duty cycle consisted of one full MS (mass spectrometry) survey scan at the mass range 400–2000 Da with a resolution power of 100,000 using the Orbitrap section. This was followed by MS2 experiments for the eight strongest peaks using the LTQ (linear trap quadrupole) section. The AGC (automated gain control) expectations during full-MS and MS/MS were 500,000 and 10,000, respectively. Intensity-based absolute quantification (iBAQ) was carried out to evaluate the protein level. Three replicates of the same sample were conducted to increase the accuracy. Protein searches were performed with the mouse protein database downloaded from the SWISS-PROT website (http://www.ebi.ac.uk/uniprot) with the following parameters: an allowance for two possible missed cleavage sites, a peptide mass tolerance of 20 ppm, and a fragment mass tolerance of 0.50 Da. Oxidized methionines and acetylated N-termini were considered as possible modifications. The acceptance criteria for protein identification were calculated to ensure that the rate of false positive identification would be less than 1%.

### Gene ontology analysis

Proteins detected at least two times were deemed valid. Differentially expressed proteins that were up-regulated (fold change >1.5) or down-regulated (fold change <0.5) in *Ildr1*^−/−^ mice were chosen for gene ontology (GO) analysis. The GO analysis was performed using the online DAVID software (http://david.abcc.ncifcrf.gov/).

## Supplementary Material

Supplementary Material
