# Supplementary material for: ILDR1 deficiency causes degeneration of cochlear outer hair cells and disrupts the structure of the organ of Corti: a mouse model for human DFNB42
Source: Biol Open. 2015 Mar 27;4(4):411–8. doi: 10.1242/bio.201410876 (PMC4400585; doi:10.1242/bio.201410876)
Supplement: Supplementary Material [file supp_4_4_411__index.html]

ILDR1 deficiency causes degeneration of cochlear outer hair cells and disrupts the structure of the organ of Corti: a mouse model for human DFNB42 — ILDR1 deficiency causes degeneration of cochlear outer hair cells and disrupts the structure of the organ of Corti a mouse model for human DFNB42 — Supplementary Material 

# ILDR1 deficiency causes degeneration of cochlear outer hair cells and disrupts the structure of the organ of Corti: a mouse model for human DFNB42

## bio.201410876 Supplementary Material

**Files in this Data Supplement:**

- Supplementary Material - Qing Sang et al. doi: 10.1242/bio.201410876
- Table S1 - Downregulated proteins in cochlea of *Ildr1*-/- mice compared with *Ildr1*+/- mice.
- Table S2 - Upregulated proteins in cochlea of *Ildr1*-/- mice compared with *Ildr1*+/- mice.
