# Supplementary material for: ILDR1 deficiency causes degeneration of cochlear outer hair cells and disrupts the structure of the organ of Corti: a mouse model for human DFNB42
Source: Biol Open. 2015 Mar 27;4(4):411–8. doi: 10.1242/bio.201410876 (PMC4400585; doi:10.1242/bio.201410876)
Supplement: Supplementary Material [file supp_bio.201410876_bio.201410876-s1.pdf]

Biology Open (2015) 000, 1–8 doi:10.1242/bio.201410876

Supplementary Material

Qing Sang et al. doi: 10.1242/bio.201410876

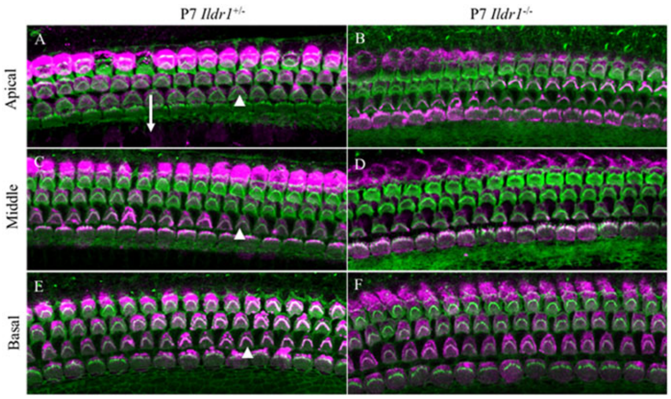

Fig. S1. Hair cells in the organ of Corti labeled with myosin 7a antibody (magenta) and counterstained with phalloidin labeling of cytoskeletal filamentous actin (green) in P7 mice. Hair cells in the apical (A,B), middle (C,D), and basal turns (E,F) of the cochleae are shown separately. Both outer (pointed by white arrowheads) and inner hair cells (indicated by white arrows) are normal and intact in *Ildr1*$^{+/-}$ and *Ildr1*$^{-/-}$ P7 mice.

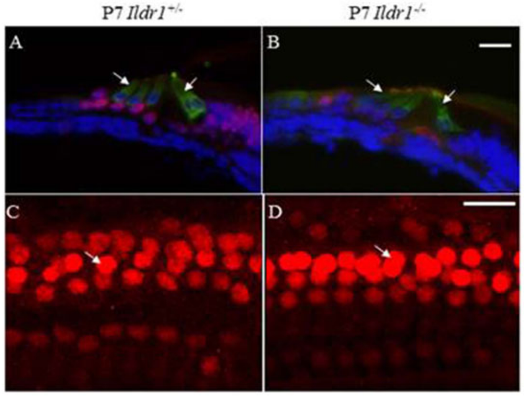

Fig. S2. Hair cells and supporting cells are normal in *Ildr1*$^{+/-}$ and *Ildr1*$^{-/-}$ P7 mice. (A,B) Hair cells were immunostained with myosin 7a antibody (green). (C,D) Supporting cells were immunostained with SOX2 antibody (red). Arrows in A,B point to the outer and inner hair cells. Arrows in C,D point to the supporting cells. Scale bars: 20 µm.

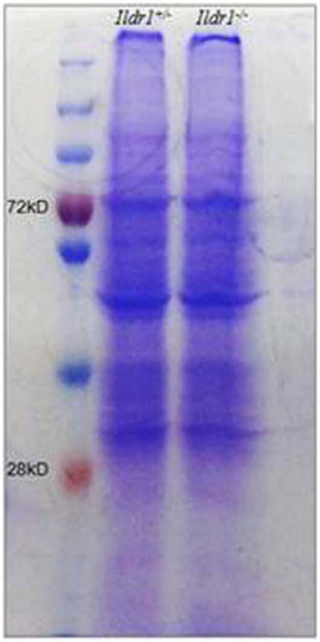

Fig. S3. Protein quality verification by SDS-PAGE and Coomassie blue staining. Proteins were extracted from *Ildr1*$^{+/-}$ and *Ildr1*$^{-/-}$ P21 mice cochleae. A total of 20 µg of protein was loaded for electrophoresis. Red bands in the marker represent 72 kDa and 28 kDa.

Tables S1 and S2: See supplementary webpage
