# Supplementary material for: ILDR1 deficiency causes degeneration of cochlear outer hair cells and disrupts the structure of the organ of Corti: a mouse model for human DFNB42
Source: Biol Open. 2015 Mar 27;4(4):411–8. doi: 10.1242/bio.201410876 (PMC4400585; doi:10.1242/bio.201410876)
Supplement: Supplementary Material [file supp_bio.201410876_Table_S1.docx]

| **Table S1. Down-regulated proteins in cochlea of *Ildr1*^-/-^ mice compared with *Ildr1*^+/-^ mice** | | | |
| --- | --- | --- | --- |
| gene symbol | iBAQ-*Ildr1*^-/-^ | iBAQ-*Ildr1^+^*^/-^ | iBAQ-*Ildr1*^-/-^/*Ildr1*^+/-^ |
| Arhgap27 | 0 | 11411.1 | 0 |
| Myh8 | 0 | 9755.366667 | 0 |
| Tnnc2 | 0 | 35959 | 0 |
| Gnl1 | 0 | 12738.66667 | 0 |
| Prrx1;Prrx2 | 0 | 65912.5 | 0 |
| Ptprg | 0 | 11707 | 0 |
| Eml6 | 0 | 61564 | 0 |
| Thnsl2 | 0 | 18550.33333 | 0 |
| Mtrf1l | 0 | 33327 | 0 |
| Eftud1 | 0 | 27102.33333 | 0 |
| Micu2 | 0 | 78680.5 | 0 |
| Dpyd | 0 | 21180 | 0 |
| Wipf1 | 0 | 39887.5 | 0 |
| Ddx52 | 0 | 10630 | 0 |
| Uap1 | 0 | 37210.66667 | 0 |
| Coa5 | 0 | 295100 | 0 |
| Cuta | 0 | 134956.6667 | 0 |
| Nudt14 | 0 | 423170 | 0 |
| Mrps33 | 0 | 177883.3333 | 0 |
| Otof | 0 | 5931.3 | 0 |
| Ocm | 192343.3333 | 1868000 | 0.102967523 |
| Anp32e | 244160 | 1857433.333 | 0.131450209 |
| Bsdc1 | 24353 | 168961.3333 | 0.144133569 |
| Commd6 | 97435 | 632880 | 0.153954936 |
| Anp32b | 133014.6667 | 855426.6667 | 0.155495114 |
| Hmgb2 | 215175 | 1381000 | 0.155811007 |
| Arg2 | 34429.66667 | 190446 | 0.180784404 |
| Tbcb | 231213.3333 | 1229546.667 | 0.188047627 |
| Dynlt1 | 248570 | 1314913.333 | 0.189039075 |
| Plin4 | 7511.5 | 33989.76667 | 0.220992985 |
| Trpv4 | 13735 | 59843.33333 | 0.229515958 |
| Stfa2 | 152760 | 656936.6667 | 0.232533831 |
| Ptges3 | 244590 | 990843.3333 | 0.246850326 |
| Supt6h | 8515.966667 | 34303.33333 | 0.248254786 |
| Arl10 | 82582.66667 | 328315.6667 | 0.251534347 |
| Itgb2l | 24272 | 94481 | 0.256898212 |
| H1f0 | 1277573.333 | 4935066.667 | 0.258876611 |
| Sart3 | 14502.33333 | 55575.66667 | 0.260947537 |
| Otud4 | 10046.6 | 34563.06667 | 0.290674439 |
| Nup85 | 833170 | 2857950 | 0.291527144 |
| Snrpa | 41223 | 138272 | 0.298129773 |
| Oscar | 1141505.667 | 3823066.667 | 0.298583772 |
| Anapc1 | 3860.233333 | 12914.36667 | 0.298909999 |
| Plekhf1 | 65926.66667 | 218210 | 0.302124864 |
| Calb1 | 189586 | 609870 | 0.310862971 |
| Sdsl | 18953.66667 | 59278.33333 | 0.319740209 |
| Rpl35a | 1618300 | 5035200 | 0.321397363 |
| Myh10 | 717596.6667 | 2213683.333 | 0.324164101 |
| Arih1 | 13352.43333 | 40701.33333 | 0.328058868 |
| Vcam1 | 355656.6667 | 1064663.333 | 0.334055523 |
| Rpl24 | 1677966.667 | 4952433.333 | 0.338816609 |
| Mrps14 | 82117 | 241070 | 0.3406355 |
| Ahcyl2 | 1183266.667 | 3459466.667 | 0.342037308 |
| Set | 423733.3333 | 1231003.333 | 0.34421786 |
| Bhmt | 92808.33333 | 269230 | 0.344717652 |
| Fndc3b | 8081.1 | 23049.33333 | 0.350600162 |
| Dnaja2 | 17326 | 49164.33333 | 0.352409945 |
| Itm2b | 671676.6667 | 1897700 | 0.353942492 |
| Nap1l4 | 678536.6667 | 1913266.667 | 0.354648246 |
| Abcb6 | 408336.6667 | 1123543.333 | 0.363436509 |
| Ufsp2 | 11269.36667 | 30867.66667 | 0.365086444 |
| Gnl3 | 25111.66667 | 68405.33333 | 0.367101006 |
| Rpl30 | 6524866.667 | 17632033.33 | 0.370057528 |
| Rangap1 | 387693.3333 | 1042240 | 0.371980862 |
| Rpl14 | 6180833.333 | 16605000 | 0.372227241 |
| Yeats4 | 108075.3333 | 287543.6667 | 0.375857116 |
| Kif3b;Kif3c | 7299.5 | 19403.33333 | 0.376198248 |
| Pdap1 | 27735.66667 | 72374 | 0.383226942 |
| Pnpla8 | 11596.45 | 29762.33333 | 0.389635109 |
| Rpl32 | 1750233.333 | 4458200 | 0.392587442 |
| Casp8 | 50264.66667 | 127409 | 0.394514255 |
| Snrpe | 2671166.667 | 6770666.667 | 0.394520481 |
| Cdan1 | 25878.63333 | 65006 | 0.398096073 |
| Naa38 | 6694333.333 | 16805166.67 | 0.398349714 |
| Nfasc | 25634.33333 | 64096 | 0.399936554 |
| Mrps35 | 20622 | 50480 | 0.408518225 |
| Supt5h | 169986.6667 | 413820 | 0.410774411 |
| Ndufs8 | 138351 | 335640 | 0.412200572 |
| Tgfb1i1 | 19063 | 46075.33333 | 0.413735477 |
| Tbcel | 91483.66667 | 217070 | 0.421447766 |
| Hsp90aa1 | 2171200 | 5112666.667 | 0.424670752 |
| Eif3d | 110874.6667 | 259343.3333 | 0.42752079 |
| Lmcd1 | 70484.33333 | 164260 | 0.429102236 |
| Rpl28 | 2085353.333 | 4856000 | 0.429438495 |
| Adhfe1 | 29723 | 68929.66667 | 0.431207656 |
| Dgke | 11579.03333 | 26822.66667 | 0.431688373 |
| Slc26a5 | 10406.25 | 24083 | 0.432099406 |
| S100a9 | 65720333.33 | 151963333.3 | 0.432474939 |
| Pik3c3 | 104035.6667 | 238799.3333 | 0.435661462 |
| Cox17 | 3604766.667 | 8235533.333 | 0.437708952 |
| Dnajc8 | 85608.66667 | 193676.6667 | 0.442018484 |
| Pmm1 | 51305.66667 | 115280 | 0.445052625 |
| Fam101b | 231710 | 520143.3333 | 0.445473363 |
| Anp32a | 793190 | 1764466.667 | 0.44953527 |
| Mrpl37 | 39205.33333 | 86649.33333 | 0.452459723 |
| Nek7 | 61569.66667 | 135650 | 0.453886227 |
| Nap1l1 | 586023.3333 | 1286166.667 | 0.45563561 |
| Rab8b | 48141.5 | 105114.3333 | 0.457991774 |
| Mcee | 213656.3333 | 465623.3333 | 0.458860882 |
| Rpl21 | 2883900 | 6280200 | 0.459205121 |
| Smarca4 | 4109.633333 | 8918.966667 | 0.460774604 |
| Rpl22l1 | 272483.3333 | 591136.6667 | 0.460948117 |
| Wdr37 | 58299.33333 | 120936.6667 | 0.482064993 |
| Tmcc2 | 14741.7 | 30520.66667 | 0.483007143 |
| Tsc1 | 7771.85 | 15937 | 0.487660789 |
| Fam63b | 54213.33333 | 110807 | 0.489259102 |
| Rnf214 | 15573.73333 | 31766.33333 | 0.490259079 |
| Mxra8 | 39196 | 79918.33333 | 0.490450668 |
| Dpp7 | 219236.6667 | 446820 | 0.490659923 |
| Txndc9 | 37581.33333 | 76189.33333 | 0.493262399 |
| Trim21 | 40368.66667 | 81675.33333 | 0.494257752 |
| Epn2 | 95212 | 191686.3333 | 0.496707294 |
| Them6 | 51306.66667 | 103228.3333 | 0.497021167 |
