# Supplementary material for: ILDR1 deficiency causes degeneration of cochlear outer hair cells and disrupts the structure of the organ of Corti: a mouse model for human DFNB42
Source: Biol Open. 2015 Mar 27;4(4):411–8. doi: 10.1242/bio.201410876 (PMC4400585; doi:10.1242/bio.201410876)
Supplement: Supplementary Material [file supp_bio.201410876_Table_S2.docx]

| **Table S2. Up-regulated proteins in cochlea of *Ildr1*^-/-^ mice compared with *Ildr1*^+/-^ mice** | | | |
| --- | --- | --- | --- |
| gene symbol | iBAQ-*Ildr1*^-/-^ | iBAQ-*Ildr1*^+/-^ | iBAQ-*Ildr1*^-/-^/*Ildr1*^+/-^ |
| Eif3m | 1780133.333 | 1185433.333 | 1.501673087 |
| Rfc2 | 85114.66667 | 56620.33333 | 1.50325266 |
| Steap3 | 1076450 | 715966.6667 | 1.503491783 |
| Ndrg1 | 11387333.33 | 7569966.667 | 1.50427787 |
| Mme | 617353.3333 | 410066.6667 | 1.505495041 |
| Ltbp4 | 17599 | 11688.66667 | 1.505646495 |
| Abcb1a;Abcb1b | 296103.3333 | 196660 | 1.505661209 |
| Sirpa | 132216.6667 | 87805.66667 | 1.505787402 |
| Gmfg | 1288933.333 | 855940 | 1.505868791 |
| Gng2 | 5949633.333 | 3947600 | 1.507152025 |
| Atg7 | 1129866.667 | 749610 | 1.507272671 |
| Hadh | 9148433.333 | 6069200 | 1.507354072 |
| Ctsc | 541340 | 358813.3333 | 1.508695329 |
| Rabac1 | 713403.3333 | 472756.6667 | 1.509028605 |
| Alox12 | 129645 | 85850.66667 | 1.510122228 |
| Lama4 | 271786.6667 | 179946.6667 | 1.510373444 |
| Pde12 | 67973 | 44980 | 1.511182748 |
| Itih1 | 479790 | 317473.3333 | 1.511276538 |
| Phex | 3735400 | 2469300 | 1.512736403 |
| Myl1;Myl3 | 1430590 | 945406.6667 | 1.513200668 |
| Twf2 | 2246933.333 | 1483100 | 1.515024835 |
| Psmc1 | 2935600 | 1935933.333 | 1.516374531 |
| Slc15a4 | 34667 | 22853 | 1.516956198 |
| Cd9 | 17982000 | 11852733.33 | 1.517118414 |
| Timm10 | 276570 | 182173.3333 | 1.518169509 |
| Smpd2 | 404926.6667 | 266703.3333 | 1.518266238 |
| Aplp2 | 360150 | 237176.6667 | 1.518488328 |
| Pdcd10 | 1496300 | 985156.6667 | 1.518844718 |
| Gfap | 3461233.333 | 2278300 | 1.519217545 |
| Smpd3 | 154320 | 101554.6667 | 1.519575664 |
| Rfc5 | 278856.6667 | 183503.3333 | 1.519627255 |
| Iars | 939103.3333 | 617113.3333 | 1.521768017 |
| Coq5 | 18148 | 11914 | 1.523249958 |
| Lpgat1 | 81120 | 53252.66667 | 1.523303997 |
| Hmox2 | 834240 | 547620 | 1.523392133 |
| App | 1200830 | 788193.3333 | 1.523522148 |
| Lcat | 49337 | 32375.33333 | 1.52390709 |
| Herc4 | 129940 | 85202.33333 | 1.525075604 |
| Syn1 | 103815.6667 | 68063.66667 | 1.525272906 |
| Ubqln4 | 68371.66667 | 44811.33333 | 1.525767291 |
| Fam162a | 1899933.333 | 1245200 | 1.525805761 |
| Asns | 204150 | 133750 | 1.52635514 |
| Eif3k | 1079950 | 707373.3333 | 1.526704428 |
| Cct6a | 8827400 | 5780000 | 1.527231834 |
| Ambp | 125433.3333 | 82074.33333 | 1.528289396 |
| Erp44 | 4637066.667 | 3033433.333 | 1.528652902 |
| Nfic | 58601.66667 | 38319.33333 | 1.529297657 |
| Marcks | 6324766.667 | 4134033.333 | 1.529926383 |
| Amph | 352146.6667 | 230046.6667 | 1.530761874 |
| Rasa3 | 148003.3333 | 96607.33333 | 1.532009302 |
| Bcas2 | 652626.6667 | 425816.6667 | 1.53264707 |
| Slc7a1 | 264330 | 172343.3333 | 1.533740789 |
| Palmd | 94818.66667 | 61815.66667 | 1.533893781 |
| Clybl | 440596.6667 | 287190 | 1.534164374 |
| Pdcd4 | 611890 | 398756.6667 | 1.534494721 |
| Xpo4 | 28662 | 18661.33333 | 1.535903115 |
| Atp6v0a1 | 475516.6667 | 309410 | 1.536849703 |
| Rasip1 | 36032.66667 | 23440 | 1.537229807 |
| Mbnl1 | 3664766.667 | 2381566.667 | 1.538804988 |
| Sphk1 | 97165 | 63130 | 1.539125614 |
| Tnpo3 | 200070 | 129936.6667 | 1.539750135 |
| Arl2bp | 423570 | 274953.3333 | 1.540515966 |
| Cplx2 | 521416.6667 | 338466.6667 | 1.540525901 |
| Snrpd1 | 14360000 | 9312566.667 | 1.542002384 |
| Tbc1d13 | 535846.6667 | 347336.6667 | 1.542729916 |
| Pald1 | 112162.3333 | 72701 | 1.542789416 |
| Atp6ap1 | 483656.6667 | 313466.6667 | 1.542928541 |
| Polr2e | 596760 | 386743.3333 | 1.54303888 |
| Rptor | 22556 | 14615.9 | 1.543250843 |
| Lman1 | 3688966.667 | 2389766.667 | 1.543651403 |
| Txnrd1 | 1680000 | 1087096.667 | 1.545400746 |
| Lamp2 | 221443.3333 | 143263 | 1.545711966 |
| Cybb | 1168340 | 755806.6667 | 1.545818596 |
| Cnot1 | 193450 | 125056.6667 | 1.546898739 |
| Emilin1 | 88225.33333 | 57017.33333 | 1.547342329 |
| Arfgap1 | 707406.6667 | 456736.6667 | 1.548828282 |
| Slc36a2 | 61570 | 39747 | 1.549047727 |
| Spcs2 | 1300266.667 | 838846.6667 | 1.550064771 |
| Eif3l | 1116923.333 | 720553.3333 | 1.550091134 |
| Smap2 | 457030 | 294826.6667 | 1.550165069 |
| Nckipsd | 37276 | 24033 | 1.551033995 |
| Myo1b | 487980 | 314503.3333 | 1.551589278 |
| Vapa | 2377433.333 | 1531680 | 1.552173648 |
| Prelp | 3364400 | 2166143.333 | 1.553175152 |
| Rnf213 | 205990 | 132571.6667 | 1.553801089 |
| Nol3 | 549716.6667 | 353746.6667 | 1.553984019 |
| Ap1m2 | 96346 | 61975 | 1.554594595 |
| Slc9a1 | 348783.3333 | 224303.3333 | 1.554962774 |
| Pigs | 369053.3333 | 237330 | 1.55502184 |
| Tmem214 | 119146.6667 | 76578.33333 | 1.5558796 |
| Pou2f1;Pou2f3;Pou2f2 | 132700 | 85244.33333 | 1.556701716 |
| Papss1 | 166913.6667 | 107160.3333 | 1.557606826 |
| Mapk8 | 27525.5 | 17670.33333 | 1.557723869 |
| Rhpn2 | 18909.26667 | 12137.96667 | 1.557861146 |
| Dsp | 9836.933333 | 6313.3 | 1.558128607 |
| Trim25 | 169166.6667 | 108543.6667 | 1.558512549 |
| Fmod | 661986.6667 | 424306.6667 | 1.56016089 |
| Fam118b | 37597.66667 | 24098.33333 | 1.560177052 |
| Thbs1 | 5917700 | 3790966.667 | 1.561000273 |
| Reps1 | 32918.66667 | 21086.33333 | 1.561137546 |
| Setd7 | 621776.6667 | 398223.3333 | 1.561376782 |
| Ubr3 | 6481.7 | 4150.9 | 1.561516779 |
| Lrrc8d | 409563.3333 | 262166.6667 | 1.562225048 |
| Synm | 182640 | 116871.6667 | 1.562739757 |
| Arfgap3 | 129169.3333 | 82613 | 1.563547303 |
| Ciapin1 | 1868800 | 1195190 | 1.563600766 |
| Stat3 | 750510 | 479720 | 1.56447511 |
| Itga2b | 301023.3333 | 192380 | 1.564732994 |
| Engase | 588193.3333 | 375726.6667 | 1.565481999 |
| Slc7a2 | 186326.6667 | 119019.6667 | 1.565511582 |
| Ptk7 | 43542 | 27802.66667 | 1.566108767 |
| Hsd17b12 | 1731033.333 | 1105146.667 | 1.566338103 |
| Hnrnpa1 | 20844666.67 | 13306666.67 | 1.566482966 |
| Rdh11 | 158579.6667 | 101208 | 1.56686889 |
| Edc3 | 37885.66667 | 24166.5 | 1.56769357 |
| Enpp4 | 135910 | 86670.66667 | 1.568119933 |
| Prkab1 | 519313.3333 | 331160 | 1.568164432 |
| Col11a1 | 863136.6667 | 550230 | 1.568683399 |
| Plgrkt | 238843.3333 | 152200 | 1.569272887 |
| Eefsec | 166843.3333 | 106275 | 1.569920803 |
| Gatc | 220005 | 140126.3333 | 1.570047505 |
| Pvrl2 | 145063.3333 | 92393.66667 | 1.570057111 |
| Echdc2 | 161326.6667 | 102675 | 1.571236101 |
| Abi1 | 509363.3333 | 324016.6667 | 1.572028188 |
| Bnip1 | 260230 | 165524 | 1.57215872 |
| Prkaa1 | 402726.6667 | 255896.6667 | 1.573786294 |
| Dcn | 2598800 | 1650533.333 | 1.574521367 |
| Ube2k | 1800866.667 | 1142706.667 | 1.575965836 |
| Dus3l | 131670.3333 | 83545.33333 | 1.576034568 |
| Cpe | 44304 | 28108.5 | 1.576178024 |
| Sparc | 7358133.333 | 4666333.333 | 1.57685549 |
| Cyfip2 | 1083649 | 687010 | 1.577340941 |
| Ppic | 3422733.333 | 2168233.333 | 1.578581641 |
| Mettl16 | 77599 | 49139 | 1.579173365 |
| Fkbp4 | 2111466.667 | 1335933.333 | 1.58051799 |
| Kpna2 | 521346.6667 | 329793.3333 | 1.580828398 |
| Wdr12 | 180696.6667 | 114302.6667 | 1.580861339 |
| Cdipt | 1870333.333 | 1182936.667 | 1.581093381 |
| Col6a1 | 492923.3333 | 311156.6667 | 1.584164462 |
| Azi2 | 22808.33333 | 14392.56667 | 1.584730081 |
| Mtmr9 | 94423 | 59574.33333 | 1.584961085 |
| Tubgcp3 | 40598 | 25589.66667 | 1.586499759 |
| Aqp1 | 9035400 | 5694833.333 | 1.586596037 |
| Gp9 | 256513.3333 | 161626.6667 | 1.58707309 |
| Nasp | 755296.6667 | 475873.3333 | 1.587180062 |
| Ikbkg | 132241 | 83294 | 1.587641367 |
| Mmp2 | 131672.6667 | 82916.33333 | 1.588018444 |
| Nfib | 101461.6667 | 63873.66667 | 1.588474123 |
| Polb | 135294.6667 | 85138 | 1.589121975 |
| Sh3gl1 | 1173143.333 | 736280 | 1.593338585 |
| Pmf1 | 26511.66667 | 16612.66667 | 1.595870621 |
| Gnas | 1834333.333 | 1149096.667 | 1.59632639 |
| Exosc2 | 132730 | 83120 | 1.596847931 |
| Slc12a7 | 263163.3333 | 164780 | 1.597058705 |
| Mrpl43 | 922336.6667 | 577356.6667 | 1.597516267 |
| Crip1 | 252280 | 157893 | 1.597790909 |
| Mtr | 66239.66667 | 41431.33333 | 1.59878192 |
| Pitpnb | 614320 | 383973.3333 | 1.599902771 |
| Eif3a | 810180 | 506233.3333 | 1.600408244 |
| Srp19 | 888203.3333 | 554500 | 1.601809438 |
| Vps53 | 64195 | 40073 | 1.601951439 |
| Rcn3 | 3359866.667 | 2097000 | 1.602225401 |
| Dpep1 | 304920 | 190179.6667 | 1.603325978 |
| Psmg3 | 674683.3333 | 420613.3333 | 1.604046472 |
| Vav1 | 116976.6667 | 72899.33333 | 1.604632873 |
| Kif2a | 209790 | 130554 | 1.606921274 |
| Atxn3 | 45663 | 28373 | 1.609382159 |
| Cmpk2 | 1117983.333 | 694593.3333 | 1.609550912 |
| Entpd2 | 78816 | 48953.66667 | 1.610012188 |
| Ahsg | 211473333.3 | 131206666.7 | 1.611757533 |
| Caskin1;Caskin2 | 9320.333333 | 5781.033333 | 1.612226188 |
| Lman2 | 1331233.333 | 825506.6667 | 1.612625781 |
| Tbk1 | 23789.33333 | 14751.66667 | 1.612653937 |
| Fut8 | 18332.5 | 11366 | 1.612924512 |
| Ldhd | 44105 | 27343 | 1.6130271 |
| Pon3 | 419590 | 260063.3333 | 1.613414681 |
| Sipa1l1 | 22395.66667 | 13880.25 | 1.613491592 |
| Trip6 | 67042.66667 | 41541.33333 | 1.613878547 |
| Nid2 | 117323.3333 | 72672 | 1.614422795 |
| Prtn3 | 3612366.667 | 2236560 | 1.61514409 |
| Chad | 794950 | 492063.3333 | 1.61554407 |
| Hmgn2 | 23706000 | 14671666.67 | 1.615767352 |
| Tuba1c | 1938733.333 | 1199176.667 | 1.616720361 |
| Sel1l | 226950 | 140356.6667 | 1.616952051 |
| Il6st | 40907 | 25271.13333 | 1.618724394 |
| Dctn6 | 382613.3333 | 236100 | 1.620556261 |
| Anapc5 | 162253.3333 | 100105.3333 | 1.620826063 |
| Rpa2 | 291463.3333 | 179790 | 1.621132061 |
| Apob | 24042.9 | 14827.7 | 1.621485463 |
| Adprm | 171196.6667 | 105512 | 1.622532666 |
| Bcan | 49294 | 30374.5 | 1.622874451 |
| Lrrc59 | 542860 | 334370 | 1.623530819 |
| Dapk2 | 149683.3333 | 92194.66667 | 1.6235574 |
| Hmgcs1 | 1061396.667 | 653626.6667 | 1.623857656 |
| Tlr3 | 21681 | 13350.9 | 1.623935465 |
| Wibg | 709783.3333 | 436306.6667 | 1.626799193 |
| Plod3 | 127773.3333 | 78417.66667 | 1.629394737 |
| Cacna2d1 | 67309.33333 | 41305.66667 | 1.629542355 |
| Hmgn5 | 537406.6667 | 329633.3333 | 1.630316513 |
| Tbc1d4 | 17668 | 10830.7 | 1.631288836 |
| Tor1aip2 | 126000.3333 | 77172.66667 | 1.632706744 |
| Myl9 | 1358366.667 | 831733.3333 | 1.633175697 |
| Erap1 | 448413.3333 | 274373.3333 | 1.634318204 |
| Arhgef2 | 202460 | 123796.6667 | 1.635423679 |
| Nenf | 325736.6667 | 199020 | 1.636703179 |
| Ikbip | 228678.6667 | 139660.3333 | 1.637391672 |
| Plcxd3 | 459576.6667 | 280446.6667 | 1.638731072 |
| Tmem48 | 57439.33333 | 35030.66667 | 1.639687131 |
| Esam | 89601.33333 | 54642.66667 | 1.639768679 |
| Gatad2b | 54815.33333 | 33428 | 1.63980296 |
| Nudt9 | 216070 | 131697.6667 | 1.640651695 |
| Creld2 | 223760 | 136342.6667 | 1.64115904 |
| Praf2 | 341156.6667 | 207683.3333 | 1.642677153 |
| Pcmtd1 | 60839.66667 | 37032.33333 | 1.642879646 |
| Ttr | 10357766.67 | 6302333.333 | 1.64348125 |
| Dynlt3 | 1067163.333 | 648446.6667 | 1.645722599 |
| Vps51 | 15275.33333 | 9280.65 | 1.645933564 |
| Ltbp3 | 28758.66667 | 17466.3 | 1.646523114 |
| Gdap1l1 | 39258.66667 | 23840.33333 | 1.646733127 |
| Sorcs2 | 8564.533333 | 5198.9 | 1.647374124 |
| Dysf | 17592.33333 | 10673.16667 | 1.648276831 |
| Dnajb1 | 415643.3333 | 251996.6667 | 1.649400124 |
| Lsm1 | 582033.3333 | 352640 | 1.650502874 |
| Nr2f2;Nr2f1 | 48819.33333 | 29562.33333 | 1.65140325 |
| Nudcd3 | 104254.6667 | 63126.66667 | 1.651515472 |
| Cadm1 | 229676.6667 | 138823.3333 | 1.654452902 |
| Commd8 | 447565 | 269830 | 1.65869251 |
| Dad1 | 7320400 | 4410266.667 | 1.659854279 |
| Rgs6 | 66936.33333 | 40320.66667 | 1.660099866 |
| Ehd4 | 1243966.667 | 749196.6667 | 1.660400696 |
| Emd | 1361233.333 | 819653.3333 | 1.660742753 |
| Ankrd13a | 241606.6667 | 145123.3333 | 1.664836805 |
| Smpd4 | 190226.6667 | 114176 | 1.666082773 |
| Dhrs7 | 932490 | 559440 | 1.666827542 |
| Postn | 2047600 | 1228150 | 1.667223059 |
| Nxn | 139543.3333 | 83614.66667 | 1.668885841 |
| Abca9 | 67821.33333 | 40627 | 1.669366021 |
| Scrn3 | 796003.3333 | 476820 | 1.669400053 |
| Ctsh | 626400 | 374496.6667 | 1.672645061 |
| Stxbp2 | 308156.6667 | 184150 | 1.673400308 |
| Gfpt1 | 255276.6667 | 152326.6667 | 1.675850147 |
| Hapln1 | 36984 | 22048.33333 | 1.6774057 |
| Klc1 | 905913.3333 | 539990 | 1.677648352 |
| Psmd7 | 3958066.667 | 2358833.333 | 1.677976401 |
| Stag1 | 18953 | 11293.33333 | 1.678246753 |
| Hmgb1 | 655296.6667 | 390130 | 1.679687967 |
| Gltp | 2474566.667 | 1473066.667 | 1.679874185 |
| Gns | 117810 | 70066.66667 | 1.681398668 |
| Rbbp9 | 5392500 | 3207066.667 | 1.681443063 |
| Bax | 1127016.667 | 670226.6667 | 1.681545547 |
| Tsr2 | 1722840 | 1024330 | 1.681918913 |
| Thumpd3 | 118474 | 70422 | 1.682343586 |
| Lyrm4 | 723796.6667 | 430183.3333 | 1.682530704 |
| Bgn | 12359666.67 | 7339066.667 | 1.684092437 |
| Lbh | 396726.6667 | 235460 | 1.684900478 |
| Ncald | 172996.6667 | 102573 | 1.68657119 |
| Col14a1 | 1951233.333 | 1156010 | 1.687903507 |
| Nfix | 167453.3333 | 99204.66667 | 1.687958228 |
| Afm | 247873.3333 | 146816.6667 | 1.688318765 |
| Trrap | 26474 | 15671.66667 | 1.689290652 |
| Zmpste24 | 1283266.667 | 759090 | 1.690532963 |
| Qsox1 | 55008 | 32526 | 1.691200885 |
| Sptlc2 | 66010.33333 | 38969.33333 | 1.693904609 |
| Elf2 | 25257 | 14901.33333 | 1.694948998 |
| Amdhd2 | 607576.6667 | 358376.6667 | 1.695357771 |
| Tbc1d23 | 118808 | 70071.33333 | 1.695529318 |
| Arfgef1 | 22612.66667 | 13334.66667 | 1.695780422 |
| Cdk2 | 97250 | 57334.66667 | 1.696181484 |
| Nhlrc2 | 276533.3333 | 162973.3333 | 1.696801113 |
| Itga11 | 34932.33333 | 20577.33333 | 1.697612259 |
| Tmem176b | 362030 | 213170 | 1.698315898 |
| Slc5a5 | 208573.3333 | 122784.3333 | 1.698696631 |
| Serping1 | 1154453.333 | 678866.6667 | 1.700559756 |
| Eif3h | 820673.3333 | 481240 | 1.705330674 |
| Cldn11 | 1413073.333 | 827920 | 1.706775212 |
| Fkbp14 | 76372.66667 | 44722 | 1.707720287 |
| Rab3il1 | 117452 | 68737 | 1.70871583 |
| Spock1;Spock3 | 33714.66667 | 19729.06667 | 1.708883002 |
| Ilkap | 51282.66667 | 30004.66667 | 1.709156353 |
| G6pc3 | 447555 | 261785 | 1.70962813 |
| Nipsnap3b | 400240 | 233998 | 1.71044197 |
| Phldb1 | 116522.6667 | 68116.66667 | 1.710633717 |
| Crp | 1034803.333 | 604610 | 1.711522028 |
| Puf60 | 1026586.667 | 599616.6667 | 1.712071601 |
| Cyp1b1 | 125678 | 73272 | 1.715225461 |
| Ifi35 | 109137 | 63627.66667 | 1.715244417 |
| Casp7 | 55926 | 32557 | 1.717787265 |
| Gatm | 281603.3333 | 163606.6667 | 1.721221629 |
| Plg | 407796.6667 | 236780 | 1.722259763 |
| Aebp1 | 347880 | 201930 | 1.722775219 |
| Parvb | 295890 | 171696 | 1.723336595 |
| Tmod3 | 1931866.667 | 1120960 | 1.723403749 |
| Olfml3 | 807083.3333 | 468250 | 1.723616302 |
| Naa25 | 125946.6667 | 73043 | 1.724281131 |
| Poglut1 | 286916.6667 | 166293.3333 | 1.725364817 |
| Arhgap31 | 35105.66667 | 20338.33333 | 1.72608375 |
| Fgfr1op2 | 131083.6667 | 75892.5 | 1.727228207 |
| Aspn | 595996.6667 | 344520 | 1.729933434 |
| Pdgfrb | 121363.3333 | 70099 | 1.731313333 |
| Fkbp7 | 140335.3333 | 81037.33333 | 1.731736813 |
| Nck1 | 136306.6667 | 78623 | 1.733674201 |
| Myo1f | 19749.66667 | 11388.33333 | 1.734201668 |
| Tmem59 | 26233 | 15123 | 1.734642597 |
| Apoe | 22654000 | 13052866.67 | 1.735557451 |
| Hspa13 | 397033.3333 | 227913.3333 | 1.742036447 |
| Serpinf1 | 7610233.333 | 4367833.333 | 1.742336017 |
| Ppib | 3121500 | 1791000 | 1.742881072 |
| Tmem109 | 1196410 | 686130 | 1.743707461 |
| Dhx29 | 59893.33333 | 34327.33333 | 1.744770931 |
| Fcgrt | 293036.6667 | 167838.3333 | 1.745946 |
| Gbp2 | 614110 | 351626.6667 | 1.746483012 |
| Ubxn1 | 1513733.333 | 866370 | 1.747213469 |
| Jund | 74047 | 42354.33333 | 1.748274478 |
| Cdk1 | 399243.3333 | 228206.6667 | 1.749481464 |
| Cntn3 | 178646.6667 | 102070 | 1.750236766 |
| Aprt | 384486.6667 | 219642 | 1.750515232 |
| Tab1 | 51345 | 29319 | 1.751253453 |
| Psme2 | 3614900 | 2063200 | 1.752084141 |
| Pard3 | 12879.06667 | 7336.9 | 1.755382609 |
| Slc25a10 | 1066693.333 | 606770 | 1.757986277 |
| Gapvd1 | 61233.33333 | 34749.66667 | 1.762127215 |
| Dock8 | 26295.33333 | 14909.83333 | 1.763623559 |
| Anln | 30158.33333 | 17098.66667 | 1.763782751 |
| Alpl | 6617466.667 | 3749500 | 1.764893097 |
| Fam103a1 | 191393.3333 | 108213.5 | 1.768664107 |
| Bcs1l | 104922 | 59320.66667 | 1.768725908 |
| Rsad2 | 38241 | 21529 | 1.776255284 |
| Qrsl1 | 40079.33333 | 22556 | 1.776881244 |
| Tubb4a | 4611966.667 | 2594466.667 | 1.777616466 |
| Eif2a | 154060.6667 | 86620 | 1.778580774 |
| Paip1 | 460246.6667 | 257986.6667 | 1.783994005 |
| Ica | 190386.6667 | 106420.3333 | 1.789006487 |
| Stambpl1 | 48253.33333 | 26955.33333 | 1.79012193 |
| Inf2 | 60833 | 33980 | 1.790258976 |
| Acp6 | 148556.6667 | 82971.33333 | 1.790457748 |
| Spc24 | 116928 | 65303.33333 | 1.790536471 |
| Rftn2 | 44472.33333 | 24826.33333 | 1.791337155 |
| Atg4b | 371413.3333 | 207280 | 1.791843561 |
| Col18a1 | 52003.33333 | 28956.33333 | 1.795922596 |
| Mrpl10 | 141490 | 78693.33333 | 1.797992206 |
| Lmf1 | 208180 | 115758.6667 | 1.798396664 |
| Copz2 | 515913.3333 | 286270 | 1.802191404 |
| Stt3b | 194193.3333 | 107749.6667 | 1.802263889 |
| Bola3 | 250243.3333 | 138630 | 1.805116738 |
| Ispd | 150810 | 83532.33333 | 1.805408684 |
| Commd7 | 206636.6667 | 114283.3333 | 1.808108502 |
| Txndc5 | 5139866.667 | 2839666.667 | 1.810024651 |
| Cd34 | 137170 | 75745.33333 | 1.810936647 |
| Stt3a | 589130 | 324830 | 1.813656374 |
| Eif3e | 749703.3333 | 413363.3333 | 1.81366675 |
| Nmes1 | 492996.6667 | 271576.6667 | 1.815313048 |
| Kdelr2;Kdelr1 | 781533.3333 | 430516.6667 | 1.81533816 |
| Irgm1 | 918380 | 505656.6667 | 1.816212582 |
| Vac14 | 184196.6667 | 101351.6667 | 1.817401457 |
| Wnk1 | 109835 | 60421.33333 | 1.81781821 |
| Cfb | 937390 | 514506.6667 | 1.821920027 |
| Mndal;Ifi205a;Ifi205b | 81312.66667 | 44598.33333 | 1.823222094 |
| Vps39 | 63505.66667 | 34770 | 1.826450005 |
| Ighg1 | 528253.3333 | 288540 | 1.83078025 |
| Ap4e1 | 7964.3 | 4337.433333 | 1.836178078 |
| Ctage5 | 156573.3333 | 85117 | 1.839507188 |
| C8b | 42706.66667 | 23211.66667 | 1.839879371 |
| Prkar1b | 12017.5 | 6525.4 | 1.841649554 |
| Bicd2 | 376323.3333 | 203876.6667 | 1.84583817 |
| Ube2l6 | 314723.3333 | 170433.3333 | 1.846606689 |
| Txnrd2 | 121816.6667 | 65943.66667 | 1.847283793 |
| H3f3a;H3f3c | 196410000 | 106253333.3 | 1.848506714 |
| Clptm1l | 89016 | 48154 | 1.848569174 |
| Zfyve20 | 45709.66667 | 24682.5 | 1.851905871 |
| Commd4 | 423130 | 228266.6667 | 1.853665304 |
| Phf5a | 302310 | 163086.6667 | 1.853676982 |
| Cep41 | 1487966.667 | 802633.3333 | 1.853856057 |
| Vcpip1 | 32987.33333 | 17721.2 | 1.861461602 |
| Sat2 | 191480 | 102825.6667 | 1.862180973 |
| Emilin2 | 634316.6667 | 340610 | 1.862296077 |
| Lrp4 | 12425.5 | 6668.866667 | 1.863210141 |
| Colgalt2 | 118657 | 63679.66667 | 1.86334204 |
| Tnfaip8l2 | 374506.6667 | 200966.6667 | 1.86352629 |
| Apmap | 1036663.333 | 555503.3333 | 1.86616942 |
| Cd63 | 1507250 | 807630 | 1.866263016 |
| Rbpms | 13831333.33 | 7405266.667 | 1.867769785 |
| Ptpra | 133410 | 71277.33333 | 1.871703019 |
| Col5a2 | 69453.66667 | 37096.33333 | 1.872251525 |
| Parp14 | 8698.433333 | 4642.1 | 1.873814294 |
| Cetn3 | 282273.3333 | 150085 | 1.88075646 |
| Aldh1a2 | 732116.6667 | 388973.3333 | 1.882177013 |
| Cox15 | 99321.33333 | 52764 | 1.882369292 |
| Exoc4 | 153516.6667 | 81464.33333 | 1.884464776 |
| Kdm5c | 596790 | 316370 | 1.886367228 |
| Hacl1 | 24249 | 12851 | 1.886934869 |
| Plscr1 | 131871.6667 | 69880 | 1.887116008 |
| Pik3r1 | 37989 | 20130.26667 | 1.887158309 |
| Smchd1 | 42544.66667 | 22532.33333 | 1.888160717 |
| Clu | 11531333.33 | 6103900 | 1.889174681 |
| Tmem119 | 544206.6667 | 288053.3333 | 1.889256619 |
| Atl2 | 64702.66667 | 34232.5 | 1.890094696 |
| Pcyox1 | 1124700 | 594950 | 1.890410959 |
| Itpr2 | 8560.733333 | 4521.866667 | 1.893185705 |
| Itgav | 359340 | 189740 | 1.893854749 |
| Vimp | 164563.3333 | 86785.33333 | 1.896211341 |
| Psmb8 | 230350 | 121390 | 1.897602768 |
| Stk11 | 60675.66667 | 31953 | 1.898903598 |
| Atp1a4 | 108570 | 57009.33333 | 1.904425006 |
| Emc2 | 971813.3333 | 510220 | 1.904694707 |
| Nlrc3 | 57307 | 30080 | 1.905152926 |
| Nlrx1 | 210476.6667 | 110353.6667 | 1.90729201 |
| Cisd3 | 271020 | 141893.3333 | 1.910026311 |
| Stk10 | 100177.5 | 52335.33333 | 1.914146593 |
| Tmx2 | 522013.3333 | 272660 | 1.914521137 |
| Atp6ap2 | 179480 | 93745.66667 | 1.914541828 |
| Ppcdc | 179243.3333 | 93488 | 1.917287067 |
| Lifr | 137900 | 71702.66667 | 1.923219964 |
| Ctnnal1 | 51611 | 26812.66667 | 1.924873816 |
| Lum | 10156733.33 | 5270266.667 | 1.92717636 |
| Ctsk | 81673 | 42308 | 1.930438688 |
| Ly6e | 809026.6667 | 418946.6667 | 1.931097037 |
| Fabp5 | 3607566.667 | 1866966.667 | 1.932314449 |
| Fetub | 445930 | 230760 | 1.932440631 |
| Gosr2 | 686375 | 355046.6667 | 1.933196575 |
| Sap18 | 31067 | 16056.5 | 1.934855043 |
| Vps11 | 40198.66667 | 20758 | 1.936538523 |
| Ceacam2;Ceacam1 | 52632.33333 | 27154 | 1.938290246 |
| Alad | 6903833.333 | 3561700 | 1.938353408 |
| Tyms | 224338.3333 | 115599.6667 | 1.940648618 |
| Stag2 | 54249 | 27913.33333 | 1.943479818 |
| Lrmp | 53057.66667 | 27253.66667 | 1.946808381 |
| Lepre1 | 199733.3333 | 102447.6667 | 1.949613298 |
| Clec3a | 89807.33333 | 46038.66667 | 1.95069362 |
| Ubfd1 | 496546.6667 | 254140 | 1.953831222 |
| Eppk1 | 57169.96667 | 29243.53333 | 1.954960983 |
| Fkbp10 | 936543.3333 | 478780 | 1.956103708 |
| Rrm2 | 188870 | 95974.66667 | 1.967915144 |
| Mocs3 | 240423.3333 | 122106.6667 | 1.968961564 |
| Pafah1b3 | 996396.6667 | 505413.3333 | 1.971449111 |
| Hmgn3 | 8876766.667 | 4501200 | 1.972088924 |
| Acss1 | 18012.2 | 9133.066667 | 1.972196269 |
| Mrpl23 | 442553.3333 | 224390 | 1.972250694 |
| Hpse | 34439 | 17399 | 1.97936663 |
| Slc38a2 | 680113.3333 | 343203.3333 | 1.98166296 |
| Spp1 | 11036333.33 | 5566433.333 | 1.982657956 |
| Prpsap2 | 521866.6667 | 263150 | 1.983152828 |
| Phka1 | 24760.66667 | 12483.3 | 1.983503294 |
| Fbln1 | 873986.6667 | 440290 | 1.985025021 |
| Sts | 111356.6667 | 56092.33333 | 1.985238624 |
| Hk2 | 116084.6667 | 58410 | 1.987410831 |
| Smarcc1 | 76638 | 38560.66667 | 1.987465639 |
| Slc13a5 | 88893.33333 | 44570 | 1.994465635 |
| Alox12l | 712290 | 356493.3333 | 1.998045779 |
| Abhd6 | 319803.3333 | 160040 | 1.998271266 |
| Hat1 | 315620 | 157943.3333 | 1.998311631 |
| Smpdl3a | 336433.3333 | 168105.3333 | 2.001324566 |
| Osgep | 119685.3333 | 59797.33333 | 2.001516233 |
| Stk4 | 509643.3333 | 254453.3333 | 2.002895095 |
| Dpp9 | 40140.33333 | 19995 | 2.007518546 |
| Abr | 31859.66667 | 15863.66667 | 2.008341913 |
| Fkbp11 | 251666.6667 | 125296.6667 | 2.008566336 |
| Tmed5 | 727716.6667 | 362286.6667 | 2.00867637 |
| Cfd | 796073.3333 | 396236.6667 | 2.009085479 |
| Naa20 | 128567.5 | 63951.5 | 2.010390687 |
| Mtx2 | 905070 | 449513.3333 | 2.013444169 |
| Myl4 | 158273.3333 | 78573.66667 | 2.014330501 |
| Siglec1 | 10909.63333 | 5404.8 | 2.01850824 |
| Akr1c13 | 28582.66667 | 14117 | 2.024698354 |
| Eya4 | 74478.66667 | 36766 | 2.025748427 |
| Pcid2 | 118080 | 58249 | 2.027159265 |
| Efemp2 | 157116.6667 | 77471.66667 | 2.028053267 |
| Cd47 | 3564333.333 | 1757366.667 | 2.028224047 |
| Ndufb3 | 3216200 | 1584000 | 2.030429293 |
| Col1a1 | 4301833.333 | 2117100 | 2.031946216 |
| Bglap2;Bglap | 254893.3333 | 125342 | 2.033582784 |
| Clec2d | 76494 | 37598.66667 | 2.034487039 |
| Cep170 | 26290 | 12901.63333 | 2.037726489 |
| Phospho1 | 724963.3333 | 355583.3333 | 2.038800094 |
| Tapbp | 480933.3333 | 235803.3333 | 2.039552735 |
| Mrpl19 | 37795 | 18447 | 2.04884263 |
| Prim2 | 58024 | 28296 | 2.05060786 |
| Nsl1 | 25267.66667 | 12277 | 2.058130379 |
| Eogt | 73500.33333 | 35691.33333 | 2.059332798 |
| Acox3 | 118696.6667 | 57545 | 2.062675587 |
| Col1a2 | 7258366.667 | 3516766.667 | 2.063931831 |
| Snap29 | 247306.6667 | 119332.6667 | 2.072413812 |
| Enpp2 | 127223.3333 | 61312 | 2.075015223 |
| Tap1 | 123000 | 59242 | 2.076229702 |
| Galnt1 | 70728 | 33985.5 | 2.081122832 |
| Podxl | 497686.6667 | 239090 | 2.081587129 |
| Slc25a24 | 101715.6667 | 48747.66667 | 2.086575084 |
| Mpp2 | 58286.66667 | 27913.66667 | 2.08810499 |
| Sirt5 | 162680 | 77630.66667 | 2.095563609 |
| Dpysl5 | 408103.3333 | 194543.3333 | 2.097750287 |
| Panx3 | 179316.6667 | 85159.33333 | 2.105660761 |
| Vps4a | 31029.5 | 14723.5 | 2.107481237 |
| Myh1 | 65067.33333 | 30842.03333 | 2.109696615 |
| Ckm | 1166880 | 552986.6667 | 2.110141293 |
| Palld | 67357.66667 | 31891.33333 | 2.112099421 |
| Akr1b7 | 181229 | 85757.66667 | 2.113268785 |
| Dkk3 | 134527.6667 | 63649.33333 | 2.113575424 |
| Ddx58 | 385073.3333 | 181493.3333 | 2.121694093 |
| Elmo2 | 120522.3333 | 56746.33333 | 2.123878782 |
| Pcsk1n | 114724.3333 | 53846.33333 | 2.13058766 |
| Ppp6r2 | 93230.33333 | 43646.33333 | 2.136040446 |
| Triobp | 15365.03333 | 7184 | 2.138785264 |
| Pyhin1 | 262720 | 121900.6667 | 2.155197401 |
| Ass1 | 225596.6667 | 104609.3333 | 2.156563468 |
| Znf148 | 43384 | 20111 | 2.157227388 |
| Cnot8 | 35915.66667 | 16632.25 | 2.159399159 |
| Map2k3 | 122032 | 56511.33333 | 2.159425248 |
| Usp24 | 136917.6667 | 63362.66667 | 2.160857077 |
| Clec3b | 5803000 | 2683966.667 | 2.162098387 |
| Tor3a | 97580 | 45071.66667 | 2.164996487 |
| Pigu | 363346 | 167691 | 2.166759099 |
| Ubxn4 | 370116.6667 | 170236.6667 | 2.174130133 |
| Wdr82 | 299646.6667 | 137803.3333 | 2.174451513 |
| Apod | 37275666.67 | 17133333.33 | 2.175622568 |
| Ankle2 | 59953 | 27469 | 2.182569442 |
| Prim1 | 53243 | 24370.83333 | 2.184701658 |
| Bclaf1 | 24302 | 11109.3 | 2.187536568 |
| Dera | 336300 | 153623 | 2.189125326 |
| Tubg1;Tubg2 | 189926.6667 | 86666.66667 | 2.191461538 |
| Mmaa | 36090.66667 | 16458.33333 | 2.192850633 |
| Fkbp9 | 866673.3333 | 394753.3333 | 2.195480722 |
| Msi1 | 377243.3333 | 171343.3333 | 2.201680836 |
| Ier3ip1 | 1663450 | 755353.3333 | 2.202214416 |
| Nubp1 | 182393.3333 | 82789 | 2.203110719 |
| Sdf4 | 184026.6667 | 83479.66667 | 2.204448988 |
| Ppap2b | 70439 | 31592.66667 | 2.229599696 |
| Tmem87a | 259340 | 116306.6667 | 2.229794795 |
| Ccdc23 | 225170 | 100856.6667 | 2.23257428 |
| Rab2b | 79663.33333 | 35570 | 2.239621404 |
| Cpsf1 | 21357 | 9529.2 | 2.241216471 |
| Tnpo2 | 177316.6667 | 78884.66667 | 2.247796361 |
| Arl6ip5 | 1931266.667 | 859016.6667 | 2.248229565 |
| Trio | 445656.6667 | 197930 | 2.251587261 |
| Prpf6 | 67418 | 29932.66667 | 2.252321878 |
| Rlbp1 | 254483.3333 | 112697.6667 | 2.258106497 |
| Pofut1 | 249580 | 110363.3333 | 2.261439488 |
| Pum2 | 53114.66667 | 23422 | 2.2677255 |
| Prps2 | 94614.33333 | 41620 | 2.273290085 |
| Hdhd3 | 179710 | 78582.33333 | 2.286900788 |
| Ncs1 | 76715.33333 | 33433.5 | 2.294564833 |
| Supv3l1 | 33203.5 | 14422.16667 | 2.302254631 |
| B2m | 430450 | 186946.6667 | 2.30252835 |
| Apom | 333203.3333 | 144533.3333 | 2.305373616 |
| Cst3 | 1938633.333 | 838326.6667 | 2.312503479 |
| Adam23 | 113440 | 48997.33333 | 2.31522804 |
| Tmem120a | 97030 | 41892 | 2.316194023 |
| Lgals9 | 590586.6667 | 254716.6667 | 2.318602369 |
| Runx2;Runx3 | 50836.66667 | 21826.66667 | 2.329108125 |
| Eif2ak2 | 177893.3333 | 76326.66667 | 2.330683903 |
| Dtd2 | 212103.3333 | 90497.33333 | 2.343752302 |
| Ndufv3 | 757566.6667 | 323005 | 2.345371331 |
| Psmb10 | 995173.3333 | 423290 | 2.351043808 |
| Psme4 | 273560 | 116169 | 2.354845096 |
| Vmac | 258950 | 109741.6667 | 2.35963247 |
| Chmp7 | 63601.33333 | 26943.66667 | 2.360529995 |
| Tpbg | 85619.66667 | 36137.66667 | 2.369263834 |
| Cdc42se2 | 739375 | 311705 | 2.372034456 |
| Ap1g2 | 19753.66667 | 8297.566667 | 2.380657783 |
| Atl1 | 58093.66667 | 24388.66667 | 2.381994369 |
| Ighg;Igh-1a | 2561933.333 | 1074846.667 | 2.383533775 |
| Clns1a | 134676.6667 | 56419.66667 | 2.387051796 |
| Tceal5;Tceal3 | 702030 | 293540 | 2.391599101 |
| Arvcf | 38874.5 | 16173 | 2.403666605 |
| Prph | 224010 | 93158.66667 | 2.404607193 |
| Stub1 | 2372433.333 | 984176.5 | 2.4105771 |
| Smtnl2 | 25107.66667 | 10409.9 | 2.411902772 |
| Athl1 | 106404.6667 | 44044.33333 | 2.415853723 |
| Oard1 | 585770 | 242406.6667 | 2.416476445 |
| Man1a2 | 19179.06667 | 7907.333333 | 2.425478459 |
| Lrch4 | 483040 | 198840 | 2.429289881 |
| Parp1 | 21539 | 8839.4 | 2.436703849 |
| Pkp4 | 114283.3333 | 46711 | 2.446604297 |
| Prmt5 | 86576 | 35255 | 2.45570841 |
| Cd79a | 154260 | 62299.33333 | 2.476109964 |
| Clec11a | 495973.3333 | 199583.3333 | 2.485043841 |
| Rbm3 | 13807333.33 | 5462166.667 | 2.527812529 |
| Trim33 | 175150 | 68832.5 | 2.544582864 |
| Ifitm3 | 1258320 | 492246.6667 | 2.556279372 |
| Lgals3 | 355120 | 138752 | 2.559386531 |
| Bag1 | 269043.3333 | 104870.3333 | 2.565485631 |
| Aif1 | 979626.6667 | 380410 | 2.575186422 |
| Tsnax | 1402166.667 | 544293.3333 | 2.576123169 |
| Acsl5 | 93539.66667 | 36203.66667 | 2.583706991 |
| Vmp1 | 167310 | 64449 | 2.596006144 |
| Isg15 | 6246800 | 2394500 | 2.608811861 |
| Rabgap1l | 40485.33333 | 15464.83333 | 2.617896518 |
| Ptplb | 2115133.333 | 801164.6667 | 2.64007316 |
| Vamp7 | 87173.33333 | 32820.66667 | 2.65605005 |
| Llgl2 | 40833.66667 | 15360 | 2.65844184 |
| Fxyd2 | 204743.3333 | 76460 | 2.677783591 |
| Srsf2 | 163506.6667 | 60851.33333 | 2.686985769 |
| Abcd4 | 15532 | 5770.766667 | 2.691496797 |
| Mtfp1 | 221403.3333 | 81778 | 2.70737036 |
| Ogfr | 19723.5 | 7273 | 2.711879555 |
| Chtop | 179526.6667 | 66093.66667 | 2.716246136 |
| C4b | 192340 | 70737 | 2.719086193 |
| Slc13a3 | 27509.33333 | 10071 | 2.731539404 |
| Cox7a2l | 373383.3333 | 136400 | 2.737414467 |
| Usp25 | 37753.33333 | 13771.16667 | 2.741476757 |
| Pam16 | 365900 | 133276 | 2.745430535 |
| Eif4ebp2 | 613286.6667 | 221680 | 2.766540358 |
| Ccny | 167273.3333 | 60306.66667 | 2.773712138 |
| Alg10b | 72364.66667 | 25962.66667 | 2.787258628 |
| Ly6a | 934930 | 334573.3333 | 2.794394851 |
| Igfbp4 | 397026.6667 | 142000 | 2.795962441 |
| Mfap4 | 550816.6667 | 196720 | 2.800003389 |
| Dnajc10 | 104085.3333 | 37056.66667 | 2.808815328 |
| Lig3 | 9450.666667 | 3348.433333 | 2.822414462 |
| Mlc1 | 80134.5 | 28267.5 | 2.834863359 |
| Hgs | 240986.6667 | 84963 | 2.836371911 |
| Qtrtd1 | 37817.33333 | 13311.9 | 2.840866693 |
| Tex10 | 12238.66667 | 4302.333333 | 2.844657938 |
| H2-K1 | 2044933.333 | 718553.3333 | 2.845903343 |
| Parp9 | 137036.6667 | 48116.66667 | 2.848008313 |
| Sardh | 32813.66667 | 11469.25 | 2.861012417 |
| Them5 | 233246.6667 | 81010.5 | 2.879215246 |
| Tars | 305810 | 105902 | 2.887669732 |
| Anxa9 | 50338 | 17387.33333 | 2.895096047 |
| Vkorc1l1 | 171130 | 58975.66667 | 2.901705223 |
| Mrpl41 | 229453.3333 | 78761.66667 | 2.913261527 |
| Paox | 489750 | 166910 | 2.934216045 |
| H2-D1;H2-L | 1286633.333 | 437510 | 2.940808972 |
| Adh1 | 169193.3333 | 57394 | 2.947927193 |
| Vamp2 | 889943.3333 | 301230 | 2.954364882 |
| Crtap | 128423.3333 | 43302.66667 | 2.965714198 |
| Rbm42 | 67361 | 22706 | 2.966660795 |
| Slc25a42 | 108946.6667 | 36703 | 2.968331381 |
| Gh1 | 1507566.667 | 507700 | 2.969404504 |
| Tmed2 | 2614100 | 872836.6667 | 2.994947508 |
| Htatsf1 | 25600.33333 | 8521.2 | 3.004310817 |
| Ero1lb | 61381.66667 | 20381.16667 | 3.011685625 |
| C1qa | 279426.6667 | 91718.33333 | 3.046573749 |
| Mylpf | 243040 | 79301 | 3.064778502 |
| Bcl2l13 | 1172420 | 382280 | 3.066914304 |
| Trex1 | 186220 | 60600.5 | 3.07291194 |
| Dtx3l | 147046.6667 | 47724 | 3.081189059 |
| Sbf1 | 20079 | 6489.633333 | 3.094011475 |
| Tuba8 | 104270 | 33695.33333 | 3.094493797 |
| Wdr6 | 45454 | 14675.66667 | 3.097235787 |
| Oas3 | 100610.6667 | 32346 | 3.110451576 |
| Ranbp1 | 7172300 | 2294673.333 | 3.125630082 |
| Aldh1l2 | 45257.66667 | 14466.13333 | 3.128525476 |
| Stat1 | 2004700 | 637823.3333 | 3.143033337 |
| Abcb8 | 38718 | 12140 | 3.189291598 |
| Slc22a2 | 64069 | 19871 | 3.224246389 |
| Pros1 | 76879 | 23829.33333 | 3.226233774 |
| Slc35f6 | 155516.6667 | 47052.66667 | 3.305161592 |
| Zbp1 | 53548.33333 | 16155.5 | 3.314557478 |
| Lgals3bp | 2025900 | 607036.6667 | 3.337360181 |
| Ccdc93 | 315136.6667 | 93142 | 3.383400256 |
| Xrcc5 | 55916.66667 | 15898.73333 | 3.517051673 |
| Aqr | 173886.6667 | 49234 | 3.53184114 |
| Trim32 | 194033.3333 | 54679.66667 | 3.548546382 |
| Impa2 | 272486.6667 | 75826 | 3.59357828 |
| Ppwd1 | 43313 | 12025.16667 | 3.60186276 |
| Rraga;Rragb | 1385556.667 | 383418.3333 | 3.613694355 |
| Ifit1 | 1213863.333 | 329120 | 3.688208961 |
| Hebp1 | 880526.6667 | 236928.6667 | 3.716420976 |
| Fam114a2 | 1186810 | 310286.6667 | 3.824882367 |
| Mov10 | 36308 | 9486.5 | 3.827333579 |
| Sil1 | 396216.6667 | 102376.3333 | 3.870197865 |
| Derl1 | 521796.6667 | 133128 | 3.919511047 |
| Gbp4 | 132060 | 33055.66667 | 3.995079008 |
| Lpcat3 | 203923.3333 | 50274 | 4.05623848 |
| Hist1h3b | 40369666.67 | 9909800 | 4.073711545 |
| Iigp1 | 26804.33333 | 6543.8 | 4.096141895 |
| Tap2 | 277436.6667 | 64921.66667 | 4.273406413 |
| Oas1a | 308770 | 72101 | 4.282464876 |
| Elovl1 | 1861733.333 | 434613.3333 | 4.283654436 |
| Acta2;Actg2 | 70893 | 16513 | 4.293162963 |
| Slc6a13 | 40059 | 9328.9 | 4.2940754 |
| Nt5e | 34019.66667 | 7763.966667 | 4.381737857 |
| Sdc3 | 338720 | 74865 | 4.524410606 |
| Tnfrsf11b | 111308 | 24348 | 4.571545918 |
| Cyp51a1 | 86011.66667 | 18517 | 4.645010891 |
| Dhx58 | 56804.66667 | 11602.63333 | 4.89584261 |
| Hist1h3a | 21490000 | 4052466.667 | 5.302943063 |
| Ifit3 | 132796.6667 | 23417.5 | 5.67083022 |
| Xaf1 | 275706.6667 | 44758 | 6.159941612 |
| Sh3glb1 | 337963.3333 | 54280.33333 | 6.226257515 |
| C5 | 22480.56667 | 3038.4 | 7.39881736 |
| Agt | 354116.6667 | 44913.66667 | 7.884385599 |
| Cdc42bpg | 77407.8 | 9038.6 | 8.564136039 |
| Atp2a1 | 55141 | 5133 | 10.74245081 |
| Hddc3 | 757013.3333 | 67208.5 | 11.26365465 |
| Add2 | 2836103.333 | 217183.3333 | 13.05856803 |
| Chst11 | 1094260 | 67627.33333 | 16.1807356 |
| Map7d1 | 105270 | 6245.4 | 16.85560573 |
| Otos | 22935000 | 973676.6667 | 23.55504736 |
| Hbb-b2 | 175823333.3 | 267563.3333 | 657.1279074 |
| Arid1a | 12025 | 0 | Not applicable |
| C2 | 20493 | 0 | Not applicable |
| Tmem55b | 68690.5 | 0 | Not applicable |
| Atxn7l3b | 163435 | 0 | Not applicable |
| Nnt | 37835.66667 | 0 | Not applicable |
| Ifit2 | 32233.66667 | 0 | Not applicable |
| Ckmt2 | 30825 | 0 | Not applicable |
| Adck5 | 13586 | 0 | Not applicable |
| Rhbdf2 | 12984.9 | 0 | Not applicable |
| Ifi44 | 40259.66667 | 0 | Not applicable |
| Inpp5b | 12885 | 0 | Not applicable |
| Iqsec1 | 27008.33333 | 0 | Not applicable |
| Hyi | 54119.33333 | 0 | Not applicable |
| Arrb2 | 50965.5 | 0 | Not applicable |
| Tktl1 | 28856 | 0 | Not applicable |
| Pecr | 108900 | 0 | Not applicable |
| Cldnd1 | 95992 | 0 | Not applicable |
| Phyhd1 | 35525 | 0 | Not applicable |
